# Synthesis, Characterization, and Intrinsic Dissolution Studies of Drug–Drug Eutectic Solid Forms of Metformin Hydrochloride and Thiazide Diuretics

**DOI:** 10.3390/pharmaceutics13111926

**Published:** 2021-11-14

**Authors:** Guadalupe Coyote-Dotor, José C. Páez-Franco, Daniel Canseco-González, Alejandra Núñez-Pineda, Alejandro Dorazco-González, Inés Fuentes-Noriega, Alfredo R. Vilchis-Néstor, Joelis Rodríguez-Hernández, David Morales-Morales, Juan Manuel Germán-Acacio

**Affiliations:** 1 Red de Apoyo a la Investigación, Coordinación de la Investigación Científica-UNAM, Instituto Nacional de Ciencias Médicas y Nutrición SZ, Ciudad de México 14000, Mexico; ibtcoyotedg@gmail.com (G.C.-D.); paez@cic.unam.mx (J.C.P.-F.); 2 CONACYT-Laboratorio Nacional de Investigación y Servicio Agroalimentario y Forestal, Universidad Autónoma de Chapingo, Texcoco de Mora 56230, Mexico; jdcanseco@conacyt.mx; 3 Centro Conjunto de Investigación en Química Sustentable CCIQS UAEM-UNAM Carretera Toluca-Atlacomulco km 14.5, Toluca 50200, Mexico; anp@unam.mx (A.N.-P.); arvilchisn@uaemex.mx (A.R.V.-N.); 4 Instituto de Química, Universidad Nacional Autónoma de México, Circuito Exterior, Ciudad Universitaria, Ciudad de México 04510, Mexico; adg@unam.mx (A.D.-G.); damor@unam.mx (D.M.-M.); 5 Laboratorio de Biofarmacia, Departamento de Farmacia, Facultad de Química, UNAM, Ciudad de México 04510, Mexico; ifuentes@unam.mx; 6 Centro de Investigación en Química Aplicada (CIQA), Boulevard Enrique Reyna Hermosillo No. 140, Saltillo 25294, Mexico; joelis.rodriguez@ciqa.edu.mx

**Keywords:** drug–drug eutectic solid forms, mechanochemical reactions, intrinsic dissolution experiments

## Abstract

The mechanochemical synthesis of drug–drug solid forms containing metformin hydrochloride (MET·HCl) and thiazide diuretics hydrochlorothiazide (HTZ) or chlorothiazide (CTZ) is reported. Characterization of these new systems indicates formation of binary eutectic conglomerates, i.e., drug–drug eutectic solids (DDESs). Further analysis by construction of binary diagrams (DSC screening) exhibited the characteristic V-shaped form indicating formation of DDESs in both cases. These new DDESs were further characterized by different techniques, including thermal analysis (DSC), solid state NMR spectroscopy (SSNMR), powder X-ray diffraction (PXRD) and scanning electron microscopy–energy dispersive X-ray spectroscopy analysis (SEM–EDS). In addition, intrinsic dissolution rate experiments and solubility assays were performed. In the case of MET·HCl-HTZ (χ_MET·HCl_ = 0.66), we observed a slight enhancement in the dissolution properties compared with pure HTZ (1.21-fold). The same analysis for the solid forms of MET·HCl-CTZ (χ_MET·HCl_ = 0.33 and 0.5) showed an enhancement in the dissolved amount of CTZ accompanied by a slight improvement in solubility. From these dissolution profiles and saturation solubility studies and by comparing the thermodynamic parameters (ΔH_fus_ and ΔS_fus_) of the pure drugs with these new solid forms, it can be observed that there was a limited modification in these properties, not modifying the free energy of the solution (ΔG) and thus not allowing an improvement in the dissolution and solubility properties of these solid forms.

## 1. Introduction

Pharmaceutical cocrystals are multicomponent crystalline entities composed of an active pharmaceutical ingredient (API) and a coformer in a defined stoichiometric ratio [1,2,3]. The API/coformer are held together by non-covalent interactions such as hydrogen bonds, π–π interactions and van der Waals forces. The benefits encountered in the preparation of pharmaceutical cocrystals have gained a lot of interest in the last years, due to the fact that the presence of the coformer in the crystal lattice of the API modifies its original physicochemical and biological properties without covalent modification [4]. Among the physicochemical modifications that can be altered are intrinsic solubility, thermal stability, dissolution rate and bioavailability [4,5].

Replacement of the coformer by another API yields the formation of drug–drug cocrystals (DDCs) [6,7], species that have emerged as an important branch in combination therapy [3,7,8,9,10,11,12,13,14,15,16,17,18]. In principle, DDCs may provide important pharmacological and clinical advantages because the therapeutic effect of two drugs can be synergistically ligated in the same solid phase [3]. The formation of DDCs depends largely on the molecular recognition between the components [19,20,21,22]. Thus, the establishment of heterosynthons (adhesive interactions) in the formation of DDCs is fundamental in the self-assembly process [21], irrespective of whether the APIs are isomorphous (size/shape similarity) [20,23]. However, sometimes when APIs have no complementarity in the functional groups and the formation of homosynthons (cohesive interactions) prevails (lack of intermolecular recognition), drug–drug solid solutions, drug–drug eutectic solids (DDESs) or simply a physical mixture (PM) can be obtained [19]. DDESs can be defined as a conglomerate of APIs that have a lower melting point compared with their parent drugs (components are immiscible in the solid state) and there is no evidence of intermolecular recognition between the constituents [24,25,26,27,28,29]. Generally, APIs in DDESs are non-isomorphous (size/shape mismatch), contrary to drug–drug solid solutions where the components have size/shape complementarity [23,24,30,31]. Besides, in drug–drug solid solutions the number of observed solid-state phases is only one, since is a continuous single-solid phase, different to DDESs where the components have an heterogeneous separation [32]. On the other hand, when drug–drug solid systems exhibit amorphous arrangements and weak molecular recognition between the constituents, they are denominated coamorphous [23,24,32,33,34,35,36]. Coamorphous systems are an amorphous continuous single solid phase, generally showing enhanced dissolution properties (spring-parachute effect) compared with their crystalline or amorphous pure APIs forms [37].

The benefits found in the preparation of DDCs [6,7], DDESs [28] or coamorphous substances [37,38] for the treatment of complex diseases have gained relevance in recent years. Due to the complexity of type 2 diabetes (T2D), administration of a single drug (monotherapy) for the management of the glycemic control seems to be inadequate since other complications can appear [39].

Thus, when monotherapy is no longer efficient for T2D, the simultaneous utilization of two or more drugs depending on the clinical profile of the patient seems to be a more appropriate approach [40,41]. Metformin hydrochloride (MET·HCl) is considered by far as the first-line drug of choice due to several benefits for the treatment of T2D, i.e., low cost and the fact that it is the only antidiabetic drug that has conclusively shown prevention in cardiovascular diseases and has provided beneficial effects on dyslipidemia and hypofibrinolysis [42]. On the other hand, thiazide drugs are referred as the diuretics of choice for the treatment of hypertension [43,44]. Despite the wide utilization of thiazide diuretics on the treatment of hypertension, their use has been related with the increase in the risk of new-onset diabetes [45].

Within the family of thiazide diuretics, chlorothiazide (CTZ) is a poor water-soluble drug (0.2 and 0.4 g/L at pH 4 and 7), exhibiting a plasma half-life of 45–120 min [46]. Furthermore, hydrochlorothiazide (HTZ) and CTZ belong to class IV according to the Biopharmaceutical Classification System (BSC), exhibiting limited oral absorption [47,48,49,50]. Hence, diverse alkaline-salts of the type NaCTZ or KCTZ [51,52] and different cocrystals of HTZ [47,48,49,53,54,55] and CTZ [56] aiming to modify the limited aqueous solubility of CTZ or HTZ have been prepared. For instance, the preparation of a DDES containing HTZ-atelonol (0.3:0.7~1 mol:2.5 mol) has been reported [24]. For this system, using intrinsic dissolution rate (IDR) experiments, it was found that the % release of HTZ in DDES HTZ-atelonol (0.3:0.7) compared with pure HTZ has a 10-fold improvement. Analogously, the formation of the coamorphous systems glicazide-HTZ and glicazide-CTZ have been reported, seeking to improve the dissolution properties of the thiazide drugs [57], however, with no noticeable difference between the dissolution rates of amorphous/crystalline HTZ and the release rate of HTZ from the coamorphous glicazide-HTZ [57]. However, similar IDR experiments showed that the coamorphous form HTZ-atelonol (0.5:0.5~1:1) has a K_int_ 12.5-fold more than HTZ crystalline and 2.2-fold better than the PM HTZ-atelonol [58].

**Scheme 1:**
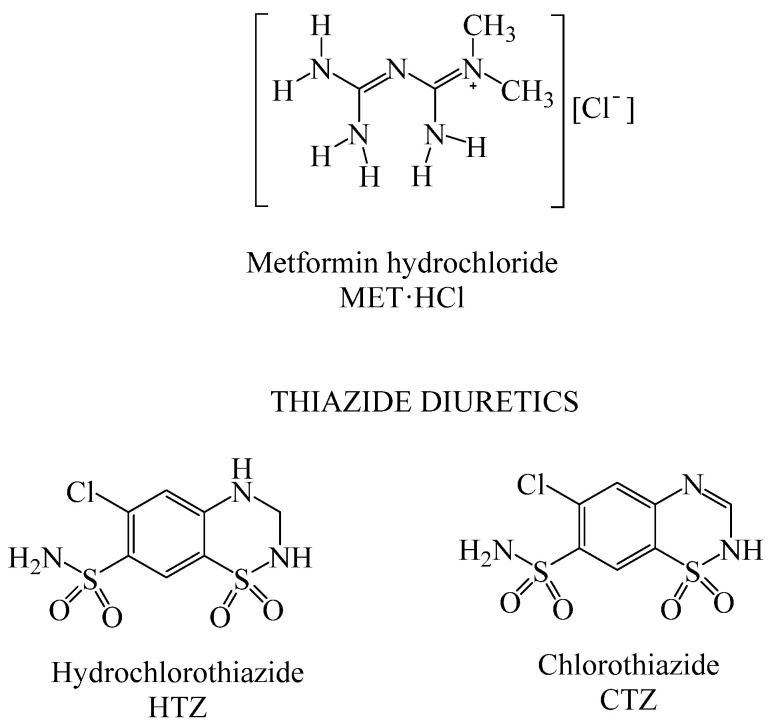
Molecular structures of MET·HCl and thiazide diuretics.

Taking this into account, we are interested in the preparation of drug–drug solid forms containing MET·HCl (classified as class III, exhibiting high solubility in water but low permeability to cell membranes) [59], in the presence of the thiazide diuretics HTZ or CTZ (Scheme 1), in order to tackle the poor solubility and limited dissolution properties of both drugs, and because many T2D patients medicated with antidiabetic drugs frequently present hypertensive complications. Thus, this paper reports the ball-milling synthesis, using neat grinding (NG) [60,61,62,63,64] or liquid-assisted grinding (LAG) [60,61,62,63,64] (varying the polarity of the solvent), and characterization [57], as well as IDR experiments to determine any modification in the dissolution properties of these solid forms compared with the pure APIs. At first, we thought we had prepared DDCs, however during characterization we discovered the formation of DDESs.

## 2. Materials and Methods

### 2.1. Materials

All the pharmaceutical reagents were purchased from Tokyo Chemical Industry America^TM^ (Portland, OR, USA) or Toronto Research Chemicals^TM^ (North York, ON, Canada) and were used as received. The solvents were purchased from Tecsiquim^TM^ (Toluca, México) and were used as received.

### 2.2. Methods

#### 2.2.1. NG or LAG Solvent-Screening (Stoichiometry Ratio 1:1)

NG or LAG solvent-screening for the preparation of the solid forms was performed using a Planetary Micro Mill Pulverisette^TM^ 7 Fritsch (Idar-Oberstein, Germany) device. MET·HCl (150.0 mg, 0.905 mmol) was ball-milled with HTZ (269.64 mg, 0.905 mmol) or CTZ (267.80 mg, 0.905 mmol) in a 1:1 stoichiometric ratio. For every LAG experiment 100 μL of solvent were added. The solvents used were, hexane, acetone, acetonitrile, and water. Stainless steel bowls of 20 mL containing 10 stainless steel balls (10 mm diameter) were used. The NG or LAG were carried out at 600 rpm for 30 min. The physical mixtures (PM) were prepared by mechanical shaking MET·HCl (150.0 mg, 0.905 mmol) with HTZ (269.64 mg, 0.905 mmol) or CTZ (267.80 mg, 0.905 mmol) in a 1:1 stoichiometric ratio for 5 min using a Vortex Genie 2 Scientific Industries^TM^ (Bohemia, NY, USA) (velocity of shaking 5).

#### 2.2.2. Thermal Analysis

A simultaneous thermal analyzer Netzsch STA 449 F3 Jupiter was used. The samples were placed (2–4 mg) in sealed non-hermetic aluminum pans and scanned at a heating rate of 10 °C/min from 30–400 °C under a dry nitrogen atmosphere.

#### 2.2.3. Eutectic Binary Mixture Screening by DSC Data

The determination of the eutectic points was made by means of construction of binary phase and Tammann diagrams [31,65]. Different DSC scans for the diverse stoichiometric compositions were prepared (1:1, 1:2, 1:3, 1:4, 1:5, 2:1, 3:1, 4:1, 5:1) to determine the eutectic points in the solid forms MET·HCl-CTZ or MET·HCl-HTZ. The different stoichiometric samples were prepared by LAG (100 μL acetonitrile) using a Planetary Micro Mill Pulverisette^TM^ 7 Fritsch at 600 rpm for 2 h. The DSC determinations proceeded at a heating rate of 10 °C/min using the thermal analyzer Netzsch STA 449 F3 Jupiter. Binary phase diagrams were constructed by plotting the melting temperatures from the different compositions (Supplementary Tables S3 and S4, SM†), considering the first endothermic event as the solidus point (T_onset_), and the second endothermic event as the liquidus (T_onset_) in function of the mole fraction of MET·HCl. Tammann diagrams were constructed by plotting ΔH_fusion_ from the different stoichiometric ratios as a function of the mole fraction of MET·HCl. Some samples were occasionally analyzed at a heating rate of 2 or 5 °C/min to improve the viewing accuracy on some thermal events.

#### 2.2.4. PXRD and Rietveld Refinements

PXRD experiments were carried out in a Bruker D8 Advance diffractometer with Bragg-Bretano geometry, Cu Kα radiation (1.54060 Å) and Linxeye detector. Each sample was measured by a continuous scan between 5–60° in 2θ, with step time 151.19°/min and step size of 0.0198°. The Rietveld refinements were carried out using the program Fullprof suite to calculate the final crystal lattices of the different outcomes [66]. Deposited CIFs in the CSD [67] were used as reference for the calculation of the Rietveld refinements of the different outcomes. For MET·HCl we used refcode JAMRIY01 corresponding to the polymorph A (Supplementary Figure S1, SM†) [68]. For CTZ we used refcode QQQAUG04 [69] and for HTZ refcode HCSBTZ [70]. In addition, PXRD and Rietveld refinements were carried out to the different eutectic binary mixtures MET·HCl-CTZ or MET·HCl-HTZ at diverse stoichiometric compositions (1:1, 1:2, 1:3, 1:4, 1:5, 2:1, 3:1, 4:1 5:1).

#### 2.2.5. Nuclear Magnetic Resonance

**Scheme 2:**
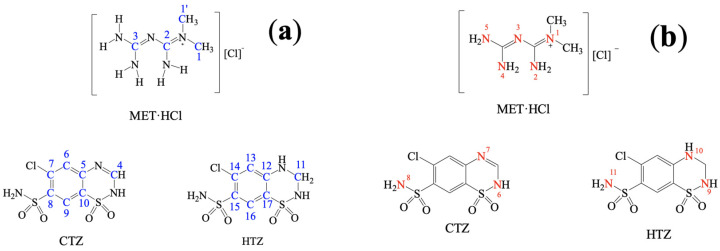
Atom numbering: (**a**) ^13^C and (**b**) ^15^N in SSNMR experiments.

Solid-state NMR (SSNMR) spectra were recorded in a Bruker Avance II 300 spectrometer (Billerica, MA, USA, operating at: ^1^H 300 MHz, ^13^C 75 MHz and ^15^N 30 MHz). SSNMR measurements were carried out on a 4 mm rotor double resonance CP-MAS probe at 5–6 kHz spinning rate with a cross-polarization contact time of 2 ms and delay of 5 s. In addition, HMBC and HSQC experiments were carried out in a Bruker Avance III 500 (operating at: ^1^H 500 MHz, ^13^C 125 MHz and ^15^N 50 MHz). Solution NMR measurements were carried out on a 4 mm broadband probe with two channels; the heteronuclear channel can be tuned ^31^P (202 MHz) until ^107^Ag (27 MHz), with *Z*-axis gradients. CTZ and HTZ were dissolved in d_6_-DMSO for the HMBC and HSQC experiments (Supplementary Figures S2–S4, SM†). The atom assignation number of the native APIs is based on Scheme 2. For the HMBC and HSQC ^15^N experiments we used NH_3(l)_ δ = 0 ppm as internal reference and glycine (δ = 38 ppm) as secondary standard. The chemical shifts assignment in the ^15^N-SSNMR experiments presented in Table 1 was made by analogy to the assignments in solution obtained from HMBC and HSQC experiments (Supplementary Figures S2–S4, SM†). In addition, the chemical shifts for the nuclei ^1^H, ^13^C and ^15^N [71,72] in d_6_-DMSO and D_2_O for MET·HCl have been previously reported [73]. In the ^15^N-SSNMR spectra for MET·HCl we noted the presence of 4 signals as well as the reported NMR solution [72]. The δ ^13^C CP-MAS assignation of CTZ [74,75,76] and HTZ [76,77,78] was on the basis of the reported data.

#### 2.2.6. Scanning Electron Microscopy Studies (SEM)-Energy-Dispersive X-ray Spectroscopy (EDS)

A JEOL (Tokyo, Japan) scanning electron microscope (SEM) model JSM-6510LV was employed to examine the morphology of the solid forms (MET·HCl-CTZ 1:1 (χ_MET·HCl_ = 0.5) and MET·HCl-HTZ 1:1 (χ_MET·HCl_ = 0.5), Supplementary Figures S9 and S10, SM†) using the secondary electron detector. Element mapping was acquired with an energy dispersive spectrometer (EDS) QUANTAX 200 from Bruker (Supplementary Figures S11 and S12, SM†). The specimens’ preparation was performed as follow: the dried samples were fixed on carbon tape over an Al-stub and finally coated with thin layer of gold using a Denton IV sputtering chamber.

#### 2.2.7. Intrinsic Dissolution Studies

We determined the IDR constants under physiological conditions. The experiments were performed using tablets, prepared with a hydraulic press at a total force of 180–200 kg/cm^2^. The dissolution rates were determined with a Wood’s apparatus according to the USP XLI. Dissolution profiles were made in 0.1 N HCl for each batch. Experiments were carried out in triplicate at 37 °C under constant stirring (50 rpm) in a constant volume of 900 mL. For CTZ and the different MET·HCl-CTZ compositions, IDR determinations were made using a HPLC Agilent 1100 with an automatic injector (DE116471) under the following chromatographic conditions: mobile phase of H_3_PO_4_ 0.025 M and acetonitrile (80:20) with a flow of 1 mL/min and using a Zorbax SB-C18 column with dimensions of 4.6 mm × 150 mm with particle size of 5 µm at a wavelength of 272 nm. For HTZ and the different MET·HCl-HTZ compositions, IDR determinations were performed using a UV-VIS spectrophotometer (Thermospectronic Helios Gamma (Waltham, MA, USA)) at the wavelength of 272 nm.

#### 2.2.8. Saturation Solubility Experiments

An excess amount of powder (eutectics or pure drug) was weighed (approximately 33.3 mg) and dissolved in a vial with a fixed volume of water (1 mL 0.1 N HCl). The vial was magnetically stirred for 72 h at 37 °C. After the equilibrium time, an aliquot was passed through a 0.45 µm filter and properly diluted and quantified through HPLC (Agilent 1260 infinity II) using a calibration curve. The experiments were made in triplicate.

## 3. Results

### 3.1. NG and LAG Solvent-Screening

#### 3.1.1. Powder X-ray Diffraction (PXRD)

The preparation of the solid forms was performed by ball-milling of MET·HCl with CTZ or HTZ (1:1) using neat grinding (NG) or liquid assisted grinding (LAG) [60,61,62,63,64]. In LAG, a solvent-screening, varying their polarity, was carried out using different solvents, such as hexane, acetone, acetonitrile and water, in order to explore their effect in the formation of new solid phases [79]. Then, all the products were analyzed by PXRD.

**Figure 1:**
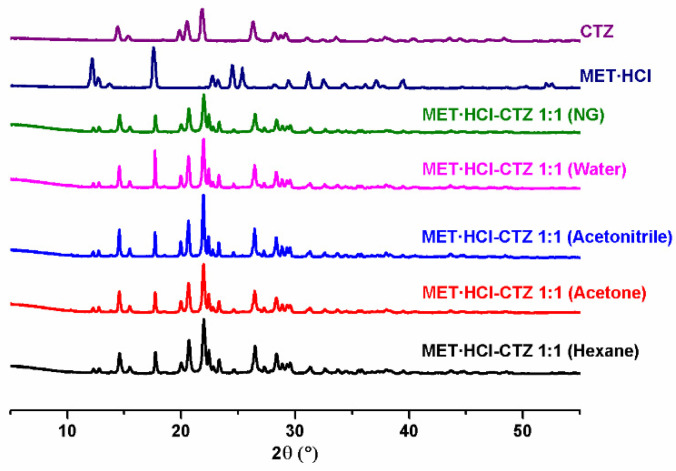
PXRD diffractograms of the parent drugs and the solid forms of MET·HCl-CTZ 1:1.

**Figure 2:**
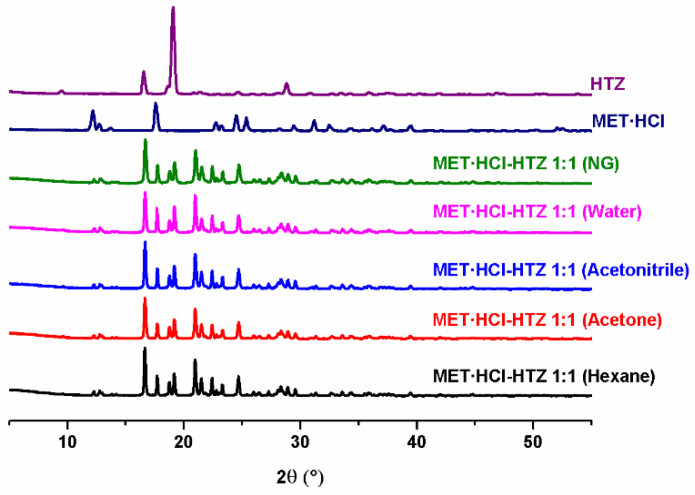
PXRD diffractograms of the parent drugs and the solid forms of MET·HCl-HTZ 1:1.

Analysis by PXRD (Figure 1 and Figure 2) of the attained solid forms of MET·HCl-CTZ 1:1 or MET·HCl-HTZ 1:1 exhibited most of the characteristic unaltered peaks of the parent APIs, suggesting the solids forms obtained were not cocrystals, and thus, Rietveld analysis was performed to determine whether new solid phases were produced [66,80].

Based on the Rietveld refinements (Supplementary Tables S1 and S2, SM†) for MET·HCl-CTZ 1:1, two crystalline lattices are observed, MET·HCl (average 31.4%, *P*2_1_/*c*) and CTZ (average 68.6%, *P*1), indicating the coexistence of two solid-phases as a conglomerate of separated components, both APIs keeping their own lattice, being more like a non-continuous single-phase than a eutectic solid. According to Cherukuvada and Nangia, “eutectic solids lack a distinct unique lattice arrangement from the individual components and retain the cohesive interactions in solid solutions” [19,20]. Similar results were obtained with the Rietveld analysis for MET·HCl-HTZ 1:1, observing MET·HCl (average 33.2%, *P*2_1_/*c*) and HTZ (average 68.6%, *P*2_1_), both constituents retaining their own lattice. Hence, no effect of the polarity of the solvent was found for the LAG solvent-screening in both solid forms, the quantitative percentage ratios for the lattice structures between the APIs remaining constant in all cases.

#### 3.1.2. Thermal Analysis

**Figure 3:**
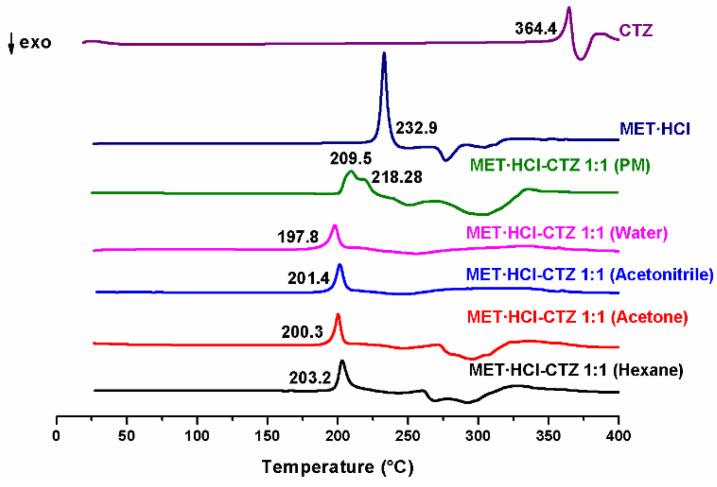
DSC scans of the parent drugs and the different products of LAG MET·HCl-CTZ 1:1.

The products obtained through the LAG solvent-screening were also analyzed by DSC. PMs were prepared combining MET·HCl with HTZ or CTZ in a 1:1 stoichiometric ratio, and analyzed by the same technique. Figure 3 shows the DSC scans of the pure APIs and those of the different outcomes for MET·HCl-CTZ 1:1, where one can easily observe the appearance of a single endothermic event, showing a considerable reduction in the T_fus_ (197.8–203.2 °C) compared with the parent APIs. In addition, for the PM the endothermic event exhibits two overlapped peaks (209.5 and 218.28 °C), clearly indicating that the different products obtained on the LAG solvent-screening are not PMs, since they only exhibit a single endothermic peak and not separated melting events for the individual components [30].

**Figure 4:**
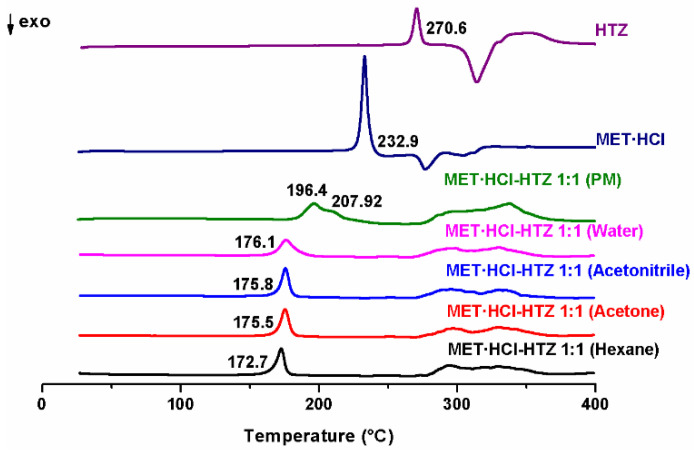
DSC scans of the parent drugs and the different products of LAG MET·HCl-HTZ 1:1.

On the other hand, the endothermic events observed in any of the products of MET·HCl-HTZ 1:1, exhibit a reduction of the T_fus_ compared with the pure MET·HCl or HTZ (Figure 4). The PM exhibits a broad peak (196.4 °C) with a shoulder (207.92 °C), considerably differing from all other endothermic events from any of the products obtained. Furthermore, as it was for the previous case, no differences were observed for the LAG-formed PMs.

#### 3.1.3. SSNMR

So far, we have seen that according to PXRD results, homosynthons predominate over the heterosynthons. In this regard, Cherukuvada and Nangia explained that “through different spectroscopical and PXRD analysis, eutectic solid phases and solid solutions have close similarity with their pure constituents” [19]. Thus, using ^13^C or ^15^N-SSNMR we can prove whether homosynthons remained intact. Thus, for the solid forms of the products prepared by LAG with acetonitrile (because the final powders were not thick) of MET·HCl-CTZ 1:1 or MET·HCl-HTZ 1:1, SSNMR experiments were performed. Atom numbering for MET·HCl, CTZ and HTZ for ^13^C and ^15^N nuclei are shown in Scheme 2.

**Figure 5:**
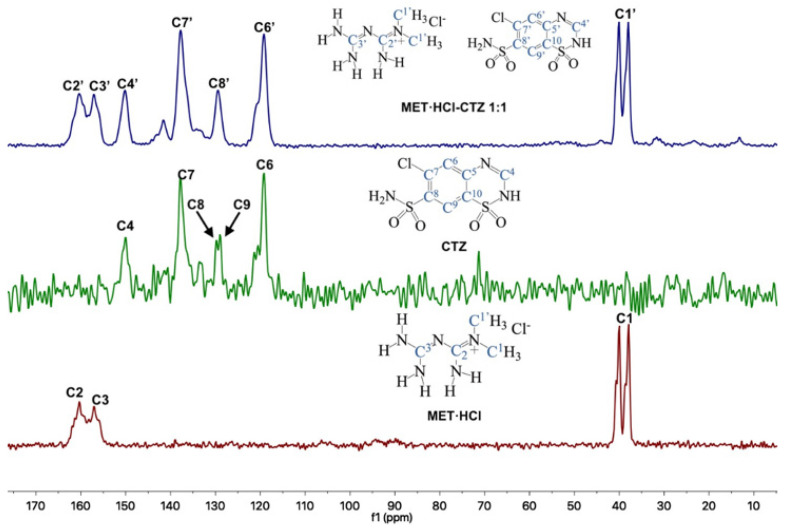
Stacked ^13^C-SSNMR spectra of MET·HCl-CTZ 1:1 (spinning rate at 6 kHz), CTZ (spinning rate at 5 kHz) and MET·HCl (spinning rate at 5 kHz).

**Figure 6:**
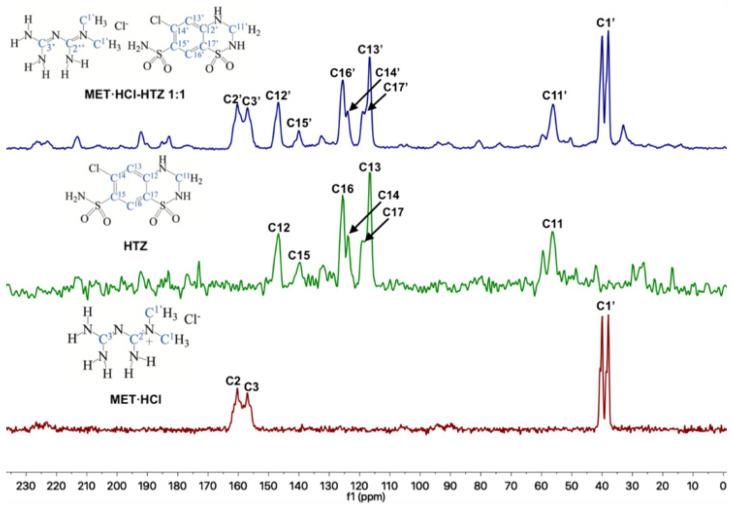
Stacked ^13^C-SSNMR spectra of MET·HCl-HTZ 1:1 (spinning rate at 5 kHz), HTZ (spinning rate at 5 kHz) and MET·HCl (spinning rate at 5 kHz).

Through ^13^C-SSNMR experiments, it can be observed that there are no significant Δδ of the pure APIs compared with the new solid forms MET·HCl-CTZ 1:1 or MET·HCl-HTZ 1:1 (Table 1). The minimal Δδ ^13^C observed for the MET·HCl-CTZ 1:1, proves that MET·HCl is not strong enough to replace the homosynthons in CTZ (Figure 5). This is probably due to the fact that CTZ has stronger intermolecular interactions, reflected in its high T_fus_ (364.4 °C) compared with MET·HCl (232.9 °C) [81]. A similar behavior is observed for MET·HCl-HTZ 1:1, where no significant Δδ ^13^C is observed compared with the original APIs (Figure 6). Once again, MET·HCl cannot replace the homosynthons in HTZ. In this regard, Haneef et al. have reported that in the DDES of HTZ:ATL (0.3:0.7) [24], the robust sulphonamide (-SO_2_NH_2_) catemer chain of HTZ cannot be interrupted by the amide group of ATL. In addition, for MET·HCl-CTZ 1:1 or MET·HCl-HTZ 1:1 apparently the non-self-complementarity (shape mismatch) of the initial components promotes the lack of formation of heterosynthons [19,21,82].

The ^13^C-SSNMR spectra of pure CTZ (Figure 5) exhibit five signals (C4, C6, C7, C8 and C9). However, Latosińska reported the appearance of only four signals due to a poorly resolved spectra (spun at 8.4 kHz) [76]. As is our case, the CP-MAS ^13^C spectrum of MET·HCl-CTZ 1:1 only showed four signals (C4′, C6′, C7′ and C8′), lacking signals for C5′, C9′ and C10′ which were not observed, probably due to lack of efficient cross polarization ^1^H-^13^C.

**Figure 7:**
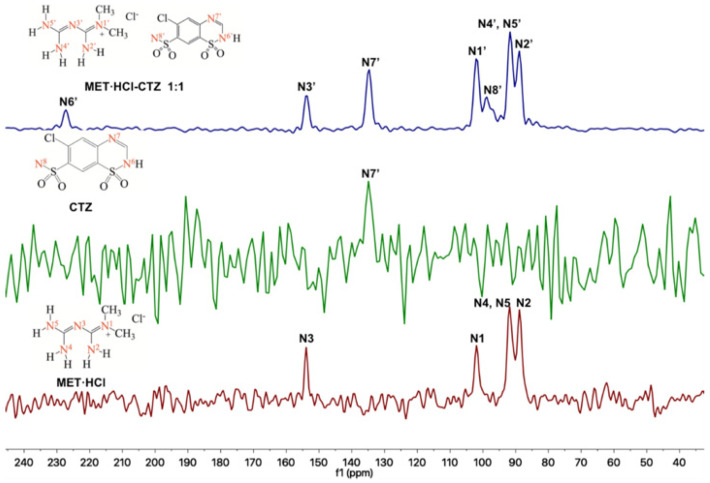
Stacked ^15^N-SSNMR spectra of MET·HCl-CTZ 1:1 (spinning rate at 6 kHz), CTZ (spinning rate at 5 kHz) and MET·HCl (spinning rate at 6 kHz).

**Figure 8:**
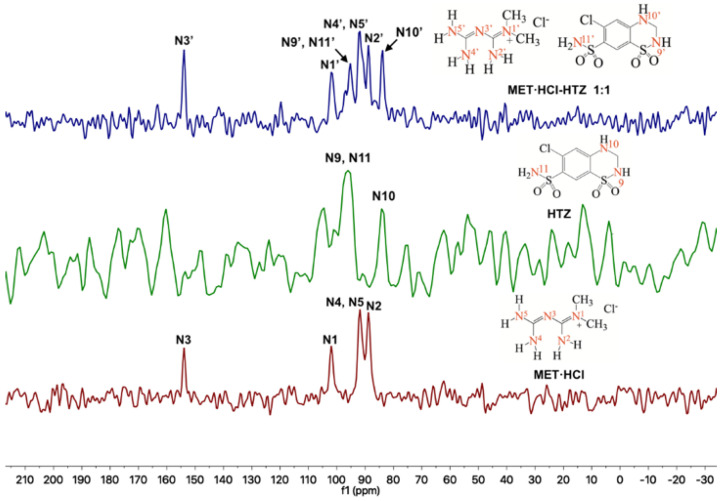
Stacked ^15^N-SSNMR spectra of MET·HCl-HTZ 1:1 (spinning rate at 5 kHz), HTZ (spinning rate at 5 kHz) and MET·HCl (spinning rate at 6 kHz).

Regarding the ^15^N-SSNMR experiments, for the APIs MET·HCl, CTZ or HTZ and the solid forms of MET·HCl-CTZ 1:1 or MET·HCl-HTZ 1:1 (Figure 7 and Figure 8), no significant Δδ were observed (Table 2). This fact indicates that there is no formation of heterosynthons and homosynthons are preserved intact.

However, in the ^15^N-SSNMR spectra of MET·HCl-CTZ 1:1 and MET·HCl-HTZ 1:1, new signals were observed that were not detected in the ^15^N-SSNMR spectra of the pure thiazide drugs. This is probably due to low-resolution spectra of the pure CTZ or HTZ due to their molecular rigidity, resulting in an inefficient cross polarization ^1^H-^15^N [81]. However, it may happen that once the solid forms are produced, the molecular rigidity of the thiazides relaxes, allowing a more efficient cross polarization ^1^H-^15^N, thus exhibiting signals for N6′ and N8′ in CTZ and N9′ and N11′ in HTZ that were unnoticeable before.

## 4. Discussion

### 4.1. Characterization of the DDESs

According to the results from DSC, PXRD and SSNMR, MET·HCl-CTZ 1:1 and MET·HCl-HTZ 1:1 are DDES forms, since the spectroscopic and diffraction data are identical to those of the original APIs and a considerable reduction of their melting points is observed [20,30]. In addition, both solid forms cannot be considered cocrystals since there is no evidence for the formation of heterosynthons [20,28]. Furthermore, Rietveld analysis proved the simultaneous residence of two crystalline lattices, thus indicating that the components are unable to form a continuous single crystalline solid [32]. Furthermore, exhaustive crystallization attempts by evaporative methods to cocrystallize both solid forms were unsuccessful, confirming that APIs are immiscible in the solid state. One of the most recurrent forms to prove the formation of DDESs is based on the construction of binary phase diagrams by DSC screening [25,30,31,83,84,85]. These binary phase diagrams can be produced from DSC scans at different compositions (1:1, 1:2, 1:3, 1:4, 1:5, 2:1, 3:1, 4:1, 5:1) [65], where some thermograms may show the appearance of two endothermic events. The first endothermic peak is considered as the solidus point (incongruent melting) and the second endothermic event as the liquidus point [65]. The appearance of these two endothermic events (solidus and liquidus points) in the DSC scans are attributed to the excess of one of the original components (non-eutectic phase) [65,83,84]. DSC scans that show no presence of the apparent liquidus point above the solidus indicate that the corresponding true eutectic composition was reached, since no evidence of unreacted or excess of reactants can be adverted. Tammann’s graphs are very helpful to confirm that a genuine eutectic composition was found in the binary phase diagrams [30,31].

**Figure 9:**
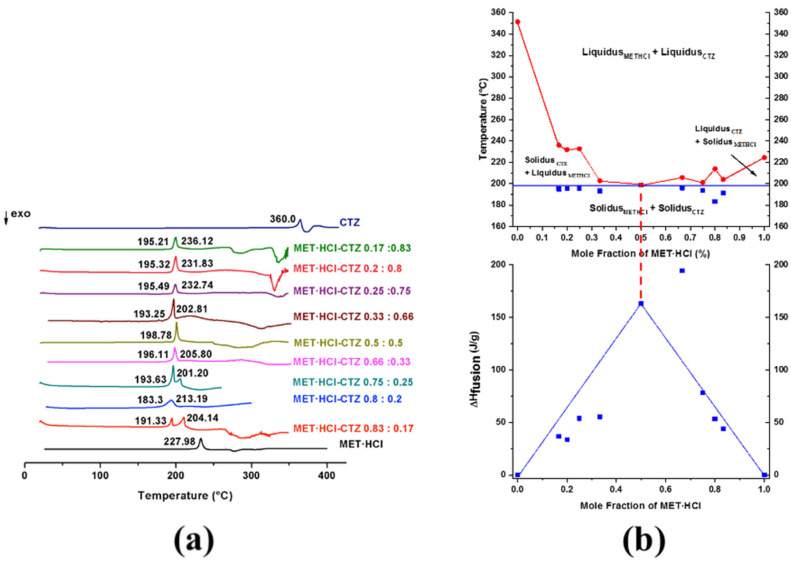
(**a**) DSC scans for MET·HCl-CTZ at different compositions (1:1, 1:2, 1:3, 1:4, 1:5, 2:1, 3:1, 4:1 5:1). (**b**) Binary phase diagram and Tammann plotting for MET·HCl-CTZ.

**Figure 10:**
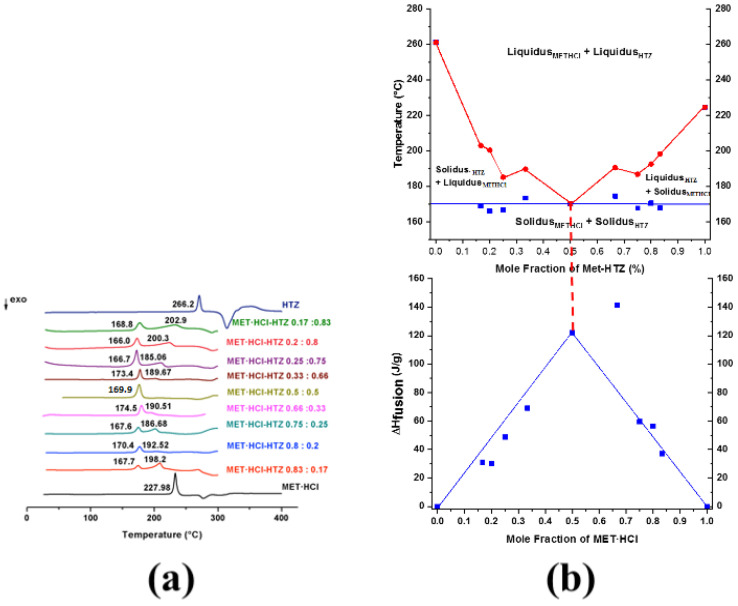
(**a**) DSC scans for MET·HCl-HTZ at different compositions (1:1, 1:2, 1:3, 1:4, 1:5, 2:1, 3:1, 4:1 5:1). (**b**) Binary phase diagram and Tammann plotting for MET·HCl-HTZ.

Thus, binary diagrams (Figure 9b and Figure 10b) were constructed, plotting the solidus and liquidus points (T_onset_) against the mole fraction of MET·HCl (χ_MET·HCl_) at all examined compositions (Supplementary Tables S3 and S4, SM†). Tammann’s triangle diagrams were plotted using the enthalpy of fusion (ΔH_fusion_) against the mole fraction of MET·HCl [30,31]. For more clarity, Figure 9a,b and Figure 10a,b can be observed in an enlarged form in the SM (Supplementary Figures S5 and S6, SM†).

The depression in the melting point, giving place to the characteristic V-shape in the binary diagram, indicates that a true eutectic composition has been found [20,64,86]. Conversely, a W-shaped graph suggests the formation of a cocrystal [20,64,86]. W-shaped graphics exhibit two eutectic points and in the middle of them resides the zone where the cocrystal exists [20,64,86].

A typical V-shaped graphic for MET·HCl-CTZ (Figure 9b) indicates that at χ_MET·HCl_ = 0.5 (molar ratio 1:1), the formation of a true eutectic composition with a T_eut_ = 198.78 °C occurs. Further, the Tammann’s triangle confirmed that at χ_MET·HCl_ = 0.5 is a true eutectic point (ΔH_fus_ = 163.3 J/g, solidus) (Supplementary Table S3, SM†), only exhibiting a single endotherm. However, for the composition χ_MET·HCl_ = 0.66 (molar ratio 2:1), a higher value (ΔH_fus_ = 194.7 J/g, solidus) was observed, with this composition not a genuine eutectic point, due to the appearance of a second peak (liquidus) caused by the presence of unreacted or excess components.

In the case of MET·HCl-HTZ (Figure 10b), the true eutectic point was found at χ_MET·HCl_ = 0.5 (molar ratio 1:1), with T_eut_ = 169.9 °C since a single endotherm was observed. Once again, according to Tammann’s triangle the highest value of ΔH_fus_ (141.4 J/g, solidus) (Supplementary Table S4, SM†) was for the composition χ_MET·HCl_ = 0.66 (molar ratio 2:1). However, this is an inaccurate determination because both endotherms (solidus and liquidus) almost form a single peak. Thus, composition χ_MET·HCl_ = 0.5 (ΔH_fus_ 122.0 J/g, solidus) gives place to the true eutectic composition.

In addition, PXRD experiments (Supplementary Figures S7 and S8, SM†) and Rietveld analysis (Supplementary Tables S5 and S6, SM†) were performed for all the different compositions examined for both MET·HCl-CTZ or MET·HCl-HTZ. Rietveld analysis for all different proportions showed the simultaneous presence of two crystalline lattices, proving that the constituents are physically separated.

Furthermore, both MET·HCl-CTZ and MET·HCl-HTZ at χ_MET·HCl_ = 0.5 were analyzed by scanning electron microscopy energy–dispersive X-ray spectroscopy SEM–EDS (Supplementary Figures S9 and S10, SM†). It has been reported that detection of light elements (C, N or O) in pharmaceutical samples can be difficult because they are not very sensitive to the EDS detector (poorer count statistics and low X-ray yields) [87,88]. For this reason, the analysis will be based on heavier atoms (Cl and S). The chemical composition acquired with EDS spectrometer for MET·HCl-CTZ (χ_MET·HCl_ = 0.5) was EDS Cl: 11.49 and S: 9.00 and calculated Cl: 15.37 and S: 13.90 (Supplementary Figure S9, SM†), whereas for MET·HCl-HTZ (χ_MET·HCl_ = 0.5) it was EDS Cl: 14.49 and S: 14.97 and calculated Cl: 15.37 and S: 13.90 (Supplementary Figure S10, SM†). All values are given in % weight.

Furthermore, the EDS mapping shows the distribution of O and S atoms in the region of interest for both MET·HCl-CTZ (χ_MET·HCl_ = 0.5) and MET·HCl-HTZ (χ_MET·HCl_ = 0.5) (Supplementary Figures S11 and S12, SM†), where it can be observed that in some zones the presence of O or S atoms is scarce, a fact that can be associated with the presence of MET·HCl molecules. This irregular distribution of O or S atoms indicates that both MET·HCl-CTZ (χ_MET·HCl_ = 0.5) or MET·HCl-HTZ (χ_MET·HCl_ = 0.5) are not a continuous single crystalline solid and the APIs are physically separated [32]. This physical separation between the components in both solid forms explain the differences between the calculated and experimental values observed in the EDS analysis. Since the intensity of the X-ray signal at any energy level is proportional to the concentration of that element in the path of the electron beam [88], when such element is reduced in concentration the maximum intensity and the peak-to-background are reduced [89].

According to the SEM micrographs, the morphology and texture observed in both solid forms are as solid phases with low crystallinity (Figures S9 and S10, SM†).

### 4.2. Microstructure Characterization

By itself, binary eutectic solids exhibit a microstructure-level periodicity different of the original pure crystalline components [90,91]. During the solidification, the effective entropy of both components changes, and this parameter can be used as an indicator to predict the microstructure adopted (Table 2). Hence, the nondimensional $\Delta {S^{0}}_{\mathrm{fus}}$, ${\Delta S}^{0}{}_{\mathrm{fus}}=\left(\frac{{\Delta H}_{\mathrm{fus}}}{T_{\mathrm{fus}}}\right)/R$ (R: gas constant) [92,93] observed with the individual components could provide a panorama of the existing micromolecular array. The values of $\Delta {S^{0}}_{\mathrm{fus}}$ can allow us to distinguish whether the solidification in the binary mixture is faceted or nonfaceted (regular) [84]. In our case, the $\Delta {S^{0}}_{\mathrm{fus}}$ values observed between MET·HCl and CTZ and between MET·HCl and HTZ are similar but greater than 2 (i.e., $\Delta {S^{0}}_{\mathrm{fus}}$ > 2 J/mol·K): MET·HCl (13.03 J/mol·K), CTZ (9.22 J/mol·K) and HTZ (7.50 J/mol·K, reported 9.61 J/mol·K) [94] (Table 2), hence both binary mixtures MET·HCl-CTZ (χ_MET·HCl_ = 0.5) and MET·HCl-HTZ (χ_MET·HCl_ = 0.5) solidified in a faceted manner (irregular microstructure) [84,93].

Furthermore, a comparison of the determined values of ΔH_fus(excess)_ with the experimental ones can be used to differentiate whether a eutectic mixture or a PM has been obtained [30]. Thus, the thermodynamic parameters were calculated according to the following expressions (χ_component_ = mole fraction of either component):

(1) $$\Delta H_{\mathrm{fusion}\left(\mathrm{excess}\right)}\;=\;\Delta H_{\mathrm{fusion}\left(\mathrm{eut}\right)\mathrm{experimental}}\;-\;\Delta H_{\mathrm{fusion}\left(\mathrm{eut}\right)\mathrm{calc}}$$

(2) $$\Delta H_{\mathrm{fusion}\left(\mathrm{calc}\right)}{\;=\;(\chi }_{\mathrm{MET}\cdot \mathrm{HCl}\;}\cdot \Delta H_{\mathrm{fusion}(\mathrm{MET}\cdot \mathrm{HCl})}{)\;+\;(\chi }_{\mathrm{thiazide}\;}\cdot \Delta H_{\mathrm{fusion}(\mathrm{thiazide})})$$

Based on the above, negative ΔH_fusion(excess)_ values were observed for both solids MET·HCl-CTZ (χ_MET·HCl_ = 0.5) and MET·HCl-HTZ (χ_MET·HCl_ = 0.5): −84.90 J/g and −110.05 J/g, thus indicating that DDESs were formed instead of PMs [30].

### 4.3. IDR Experiments

**Figure 11:**
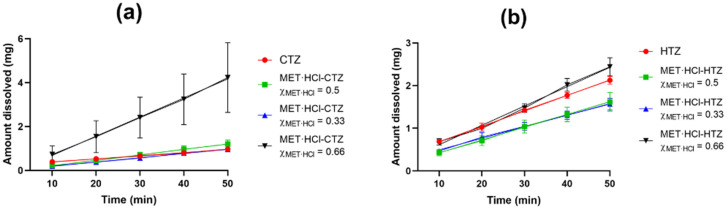
IDR experiments for the solid forms at different compositions: (**a**) MET·HCl-CTZ and (**b**) MET·HCl-HTZ. Error bars represent the standard deviation.

Due to the fact that CTZ and HTZ are the limited-water soluble drugs, in this study we only focused on the K_int_ values for both thiazides. Thus, IDR experiments were carried out only for the solid phases MET·HCl-CTZ and MET·HCl-HTZ with compositions of 1:1 (χ_MET·HCl_ = 0.5), 1:2 (χ_MET·HCl_ = 0.33) and 2:1 (χ_MET·HCl_ = 0.66), since these proportions are closer to the true eutectic points determined. Table 3 shows the K_int_ values found and Figure 11 displays the plots of the IDR experiments.

The K_int_ value determined (0.076 mg/min·cm^2^) is close to that reported for crystalline HTZ (0.098 mg/min·cm^2^) [58]. In a previous work we reported the K_int_ value of MET·HCl [73]. Specifically, the composition of MET·HCl-HTZ χ_MET·HCl_ = 0.66 showed an increase of 1.21-fold compared with pure HTZ, however the compositions χ_MET·HCl_ = 0.33 and 0.5 exhibited a decrease in the dissolution rate (0.76- and 0.77-fold). This decrease in the release behavior of HTZ can be attributed to the great excess of HTZ compared with MET·HCl, limiting any probable enhancement during dissolution [95]. In addition, the compositions MET·HCl-CTZ χ_MET·HCl_ = 0.33 and 0.5 showed an increase in the amount of CTZ dissolved (1.31 and 1.65-fold) compared with pure CTZ. However, in the case of MET·HCl-CTZ χ_MET·HCl_ = 0.66, it exhibited an increase of 6.06-fold. This should be taken with caution, since this K_int_ value presented a high standard deviation (Table 3). This is attributed to poor wettability and dispersibility of the solid form, as pronounced clumping occurred during the dissolution testing, limiting the reproducibility in triplicate determinations. Furthermore, the aqueous solubility and dissolution profile of a given compound can be quantitatively related to its T_fus_ and ΔH_fus_ [96]. Usually substances with high values of T_fus_ and ΔH_fus_ have strong intermolecular interactions and possess low solubility [95,97]. Thus, if the formation of a binary eutectic solid leads to a modification in the thermodynamic functions representing an increase in the entropy of the mixture, the enthalpy of the mixture is also modified favorably, and the alteration of these factors may contribute to increase the negative value of the free energy of solution (ΔG), overall improving the solubility of a substance [30]. Globally, the solid forms of MET·HCl-CTZ or MET·HCl-HTZ with the compositions χ_MET·HCl_ = 0.33, 0.5 and 0.66 exhibited a decrease or a modest enhancement in the amount dissolved of the thiazide drugs. Considering the thermodynamic parameters (T_fus_, ΔH_fus_ and ΔS_fus_, Table 2), in all cases there was a decrease in the T_fus_ compared with the pure constituents. However, in both these solid forms at compositions of χ_MET·HCl_ = 0.5 and 0.66, an increase in the ΔS_fus_ (increase in randomization) was observed, suggesting an enhancement in the amount dissolved of the thiazides. However, there was also a slight increase in the ΔH_fus_ compared with the original components, which affected the ΔG, leading to a limited modification in the amount dissolved, because ΔG tends to be more positive. In the case of the solid forms with composition χ_MET·HCl_ = 0.33, both parameters remain almost unchanged, reflecting a limited modification in the amount dissolved: ΔH_fus_ (limited modification in the intermolecular interactions) and ΔS_fus_ (poor dispersibility). On the other hand, in the case of the DDES HTZ:atelonol (0.3:0.7), a 10-fold improvement of the % release of HTZ (phosphate buffer and pH = 7.4) was observed [24]. Haneef et al. attribute this improvement in dissolution rate to the fact that in the eutectic phase both components established weak intermolecular interactions. In our case, a similar analysis of the ΔH_fus_ values for both solid forms MET·HCl-CTZ and MET·HCl-HTZ led us to conclude that a limited modification in the intermolecular interactions occurred after the solidification of the DDESs compared with their parent components.

According to the saturation solubility studies (Table 3), all the combinations for both solid forms exhibited an increase (the amount of drug dissolved in a saturated solution) compared with the pure components [98]. In this case, MET·HCl-CTZ χ_MET·HCl_ = 0.33, 0.5 and 0.66 improved 1.51-, 1.48- and 1.44-fold respectively, and for MET·HCl-HTZ χ_MET·HCl_ = 0.33, 0.5 and 0.66 increases in the solubility were 1.11-, 1.13- and 1.36-fold, respectively. Considering again the DDES HTZ:atelonol (0.3:0.7), an improvement in the solubility of HTZ by 14-fold was reported. The poor modifications in the solubility observed in the solid forms MET·HCl-CTZ χ_MET·HCl_ = 0.33, 0.5 and 0.66 and MET·HCl-HTZ χ_MET·HCl_ = 0.33, 0.5 and 0.66 is attributed to the limited alteration of ΔG in the solution.

## 5. Conclusions

Thus, this paper describes the mechanochemical preparation of the DDESs MET·HCl-CTZ and MET·HCl-HTZ by NG or LAG. Analyses by means of binary diagrams (DSC screening) allowed the determination of the true eutectic points at the composition of χ_MET·HCl_ = 0.5 for both solid forms. In general, it has been reported that DDESs have a lower melting point than their original components, and that these solid forms have high free energy, greater molecular mobility and weaker intermolecular interactions [19,91,93,99]. These attributes may represent a substantial improvement in their aqueous solubility and dissolution properties. However, the solid forms described in this work exhibited a limited modification in these properties, this being due to the lack of or no significant alteration in the thermodynamic parameters ΔH_fus_ and ΔS_fus_, compared with the original constituents, despite the fact that a considerable decrease in T_fus_ values is observed.

Hence, this study represents a thorough characterization of two DDESs containing MET·HCl and CTZ or HTZ. This shows that despite an active research history describing organic solid eutectics [100], when related to pharmaceutical solid eutectics they still remain partially unexplored since the analytical methods available for full characterization and understanding of their molecular and structural integrity are almost incipient compared with cocrystals. Thus, we believe that the lack of proper understanding has hampered the potential applications of these solid forms [28]. Furthermore, we believe that this study sheds further light to better comprehend the behavior of these fascinating species.

## Figures and Tables

**Table 1:** ^13^C and ^15^N-SSNMR chemical shifts (δ) of pure components and the solid forms of MET·HCl-CTZ 1:1 and MET·HCl-HTZ 1:1.

Pure APIs or New Product Solid Forms	Atom Numbering ^13^C	δ (ppm) 13C-SSNMR ^c^	Δδ (ppm) ^13^C-SSNMR	Atom Numbering ^15^N	δ (ppm) ^15^N-SSNMR ^a,b,c^	Δδ (ppm) ^15^N-SSNMR
**MET·HCl**	C1 C2 C3	37.94 and 40.01 160.29 157.02	- - -	N1 N2 N3 N4 N5	101.92 88.80 153.92 91.88 91.88	- - - -
**CTZ**	C4 C5 C6 C7 C8 C9 C10	150.02 N.D. 119.30 137.78 128.95 129.75 N.D.	- - - - - - -	N6 N7 N8	(226.95) N.D. (133.67) 134.95 (92.87) N.D.	- - -
**HTZ**	C11 C12 C13 C14 C15 C16 C17	56.33 146.76 116.59 123.82 139.71 125.49 118.98	- - - - - - -	N9 N10 N11	(91.67) N.D. (80.81) 84.08 (93.69) N.D.	- - -
**MET·HCl-CTZ 1:1**	C1′ C2′ C3′ C4′ C5′ C6′ C7′ C8′ C9′ C10′	38.00 and 40.06 160.41 157.05 150.15 N.D. 119.42 137.63 129.42 N.D. N.D.	0.06 and 0.05 0.12 0.03 0.13 - 0.12 −0.15 0.47 - -	N1′ N2′ N3′ N4′ N5′ N6′ N7′ N8′	101.91 88.90 153.80 91.66 91.66 227.28 134.79 98.93	−0.01 0.1 −0.12 −0.22 −0.22 - −0.16 -
**MET·HCl-HTZ 1:1**	C1″ C2″ C3″ C11′ C12′ C13′ C14′ C15′ C16′ C17′	37.95 and 40.01 160.28 156.98 56.16 146.78 116.65 123.96 140.03 125.52 118.89	0.01 and 0.0 −0.01 −0.04 −0.17 0.02 0.06 0.14 0.32 0.03 −0.09	N1′ N2′ N3′ N4′ N5′ N9′ N10′ N11′	101.78 88.79 153.94 92.00 92.00 95.27 83.92 97.00	−0.14 −0.01 0.02 0.12 0.12 - −0.16 -

^a^ In parenthesis are the δ established by HSQC and HMBC experiments in DMSO-d_6_. ^b^ On ^15^N-SSNMR only the chemical shift of N7 (CTZ) and N10 (HTZ) can be established. ^c^ N.D. (δ not determined) due to low-resolution of the ^13^C and ^15^N-SSNMR spectra.

**Table 2:** Experimental values of thermodynamic functions: T_fus_, ΔH_fus_, ΔS_fus_ and ΔS^0^_fus_.

Pure APIs and New Solid Forms	T_fus_ (°C)	T_fus_ (K)	ΔH_fus_ J/g	ΔH_fus_ KJ/mol	ΔS_fus_ J/mol·K	Nondimensional ΔS^0^_fus_ (ΔS_fus_/R)
MET·HCl	232.90	506.05	331.2	54.85	108.40	13.03
CTZ	364.40	637.55	165.4	48.91	76.71	9.22
HTZ	290.00	563.15	118.1	35.16	62.44	7.50
MET·HCl-CTZ χ_MET·HCl_ = 0.5	198.78	471.95	163.3	75.33	159.62	19.19
MET·HCl-HTZ χ_MET·HCl_ = 0.5	170.10	443.25	114.6	53.09	119.79	14.40
MET·HCl-CTZ χ_MET·HCl_ = 0.33	193.25	466.40	55.49	42.01	90.07	10.83
MET·HCl-HTZ χ_MET·HCl_ = 0.33	173.40	446.55	69.03	52.53	117.65	14.15
MET·HCl-CTZ χ_MET·HCl_ = 0.66	196.11	469.25	194.70	122.06	260.13	31.28
MET·HCl-HTZ χ_MET·HCl_ = 0.66	174.50	447.65	141.4	88.93	198.67	23.89

**Table 3:** K_int_ determined for both thiazide drugs and for the compositions MET·HCl-CTZ (χ_MET·HCl_ = 0.33, 05 and 0.66) and MET·HCl-HTZ (χ_MET·HCl_ = 0.33, 05 and 0.66) and saturation solubility studies.

K_int_ mg/min·cm^2^	MET·HCl-CTZ χ_MET·HCl_ = 0.5	MET·HCl-HTZ χ_MET·HCl_ = 0.5	MET·HCl-CTZ χ_MET·HCl_ = 0.33	MET·HCl-HTZ χ_MET·HCl_ = 0.33	MET·HCl-CTZ χ_MET·HCl_ = 0.66	MET·HCl-HTZ χ_MET·HCl_ = 0.66
**MET·HCl (1.600 ± 0.01)**	-	-	-	-	-	-
**CTZ (0.029 ± 0.0002)**	0.048 ± 0.007	-	0.038 ± 0.01	-	0.176 ± 0.062	-
**HTZ (0.076 ± 0.003)**	-	0.059 ± 0.007	-	0.058 ± 0.01	-	0.092 ± 0.01
Saturation Solubility (mg/mL) *	0.040 ± 0.018	0.50 ± 0.024	0.041 ± 0.006	0.49 ± 0.025	0.039 ± 0.006	0.60 ± 0.034

Associated standard deviation value for each K_int_. * SS: CTZ (0.027 mg/mL)-HTZ (0.44 mg/mL). Each value has its associated standard deviation.

## Data Availability

No new data were created or analyzed in this study. Data sharing is not applicable to this article.

## Supplementary Materials

The following are available online at https://www.mdpi.com/article/10.3390/pharmaceutics13111926/s1, Table S1: Rietveld refinements (by NG or LAG solvent-screening) for MET·HCl-CTZ 1:1, Table S2: Rietveld refinements (by NG or LAG solvent-screening) for MET·HCl-HTZ 1:1, Table S3: Thermodynamic parameters for the construction of the of binary phase and Tammann’s triangle diagram for MET·HCl-CTZ, Table S4: Thermodynamic parameters for the construction of the binary phase and Tammann’s triangle diagram for MET·HCl-HTZ, Table S5: Rietveld refinements for the different compositions for the solid form MET·HCl-CTZ, Table S6: Rietveld refinements for the different compositions for the solid form MET·HCl-HTZ, Figure S1: Rietveld refinement JAMRIY0150 (MET·HCl polymorph A) vs. MET·HCl (in blue) purchased from Tokyo Chemical Industry™, Figure S2: HSQC ^1^H-^15^N CTZ in d_6_-DMSO, Figure S3: HMBC ^1^H-^15^N CTZ in d_6_-DMSO, Figure S4: HSQC ^1^H-^15^N HTZ in d_6_-DMSO, Figure S5: Enlarged images of Figure 9a,b, Figure S6: Enlarged images of Figure 10a,b, Figure S7: PXRD experiments for the different compositions for the solid form MET·HCl-CTZ, Figure S8: PXRD experiments for the different compositions for the solid form MET·HCl-HTZ, Figure S9: SEM–EDS elemental composition analysis for the solid form MET·HCl-CTZ 1:1 (χ_MET·HCl_ = 0.5), Figure S10: SEM–EDS elemental composition analysis for the solid form MET·HCl-HTZ 1:1 (χ_MET·HCl_ = 0.5), Figure S11: EDS mapping via SEM images for MET·HCl-CTZ 1:1 (χ_MET·HCl_ = 0.5), Figure S12: EDS mapping via SEM images for MET·HCl-HTZ 1:1 (χ_MET·HCl_ = 0.5).

## References

[1] Shan N., Zaworotko M.J. (2008). The role of cocrystals in pharmaceutical science. Drug Discov. Today.

[2] Duggirala N.K., Perry M.L., Almarsson Ö., Zaworotko M.J. (2016). Pharmaceutical cocrystals: Along the path to improved medicines. Chem. Commun..

[3] Nangia A., Bolla G. (2016). Pharmaceutical cocrystals: Walking the talk. Chem. Commun..

[4] Schultheiss N., Newman A. (2009). Pharmaceutical cocrystals and their physicochemical properties. Cryst. Growth Des..

[5] Schultheiss N., Henck J.-O. (2012). Chapter 6 Role of Co-crystals in the Pharmaceutical Development Continuum. Pharmaceutical Salts and Co-Crystals.

[6] Singh Sekhon B. (2012). Drug-drug co-crystals. DARU J. Pharm. Sci..

[7] Thakuria R., Sarma B. (2018). Drug-Drug and Drug-Nutraceutical Cocrystal/Salt as Alternative Medicine for Combination Therapy: A Crystal Engineering Approach. Crystals.

[8] Wang J.-R., Yu Q., Dai W., Mei X. (2016). Drug-drug co-crystallization presents a new opportunity for the development of stable vitamins. Chem. Commun..

[9] Gopi S.P., Ganguly S., Desiraju G.R. (2016). A Drug–Drug Salt Hydrate of Norfloxacin and Sulfathiazole: Enhancement of in Vitro Biological Properties via Improved Physicochemical Properties. Mol. Pharm..

[10] Thipparaboina R., Kumar D., Chavan R.B., Shastri N.R. (2016). Multidrug co-crystals: Towards the development of effective therapeutic hybrids. Drug Discov. Today.

[11] Bhatt P.M., Azim Y., Thakur T.S., Desiraju G.R. (2009). Co-Crystals of the Anti-HIV Drugs Lamivudine and Zidovudine. Cryst. Growth Des..

[12] Aitipamula S., Chow P.S., Tan R.B.H. (2009). Trimorphs of a pharmaceutical cocrystal involving two active pharmaceutical ingredients: Potential relevance to combination drugs. CrystEngComm.

[13] Grobelny P., Mukherjee A., Desiraju G.R. (2011). Drug-drug co-crystals: Temperature-dependent proton mobility in the molecular complex of isoniazid with 4-aminosalicylic acid. CrystEngComm.

[14] Évora A.O.L., Castro R.A.E., Maria T.M.R., Rosado M.T.S., Silva M.R., Beja A.M., Canotilho J., Eusébio M.E.S. (2011). Pyrazinamide-diflunisal: A new dual-drug Co-crystal. Cryst. Growth Des..

[15] Veverka M., Šimon P., Gallovič J., Jorík V., Veverková E., Dubaj T. (2012). Imatinib mesylate cocrystals: Synthesis, screening, and preliminary characterization. Mon. Chem..

[16] Sekhon B.S. (2009). Pharmaceutical co-crystals—A review. Ars Pharm..

[17] Pinto Vitorino G., Sperandeo N.R., Caira M.R., Mazzieri M.R. (2013). A Supramolecular Assembly Formed by Heteroassociation of Ciprofloxacin and Norfloxacin in the Solid State: Co-Crystal Synthesis and Characterization. Cryst. Growth Des..

[18] Putra O.D., Furuishi T., Yonemochi E., Terada K., Uekusa H. (2016). Drug-Drug Multicomponent Crystals as an Effective Technique to Overcome Weaknesses in Parent Drugs. Cryst. Growth Des..

[19] Cherukuvada S., Nangia A. (2014). Eutectics as improved pharmaceutical materials: Design, properties and characterization. Chem. Commun..

[20] Cherukuvada S., Guru Row T.N. (2014). Comprehending the formation of eutectics and cocrystals in terms of design and their structural interrelationships. Cryst. Growth Des..

[21] Stoler E., Warner J.C. (2015). Non-Covalent derivatives: Cocrystals and eutectics. Molecules.

[22] Healy A.M., Worku Z.A., Kumar D., Madi A.M. (2017). Pharmaceutical solvates, hydrates and amorphous forms: A special emphasis on cocrystals. Adv. Drug Deliv. Rev..

[23] Allu S., Suresh K., Bolla G., Mannava M.K.C., Nangia A. (2019). Role of hydrogen bonding in cocrystals and coamorphous solids: Indapamide as a case study. Cryst. Eng. Comm..

[24] Haneef J., Chadha R. (2017). Drug-Drug Multicomponent Solid Forms: Cocrystal, Coamorphous and Eutectic of Three Poorly Soluble Antihypertensive Drugs Using Mechanochemical Approach. AAPS Pharm. Sci. Tech..

[25] Srivastava A., Zode S.S., Pandey J., Srivastava K., Tandon P., Ayala A.P., Bansal A.K. (2019). A novel approach to design febuxostat-salicylic acid eutectic system: Evaluation and characterization. CrystEngComm.

[26] Górniak A., Wojakowska A., Karolewicz B., Pluta J. (2011). Phase diagram and dissolution studies of the fenofibrate-acetylsalicylic acid system. J. Therm. Anal. Calorim..

[27] Sakata Y., Tanabe E., Sumikawa T., Shiraishi S., Tokudome Y., Otsuka M. (2007). Effects of solid-state reaction between paracetamol and cloperastine hydrochloride on the pharmaceutical properties of their preparations. Int. J. Pharm..

[28] Hoang Pham U.G. (2014). Pharmaceutical Applications of Eutectic Mixtures. J. Dev. Drugs.

[29] Carolina Bazzo G., Ramos Pezzini B., Karine Stulzer H. (2020). Eutectic mixtures as an approach to enhance solubility, dissolution rate and oral bioavailability of poorly water-soluble drugs. Int. J. Pharm..

[30] Chadha K., Karan M., Chadha R., Bhalla Y., Vasisht K. (2017). Is Failure of Cocrystallization Actually a Failure? Eutectic Formation in Cocrystal Screening of Hesperetin. J. Pharm. Sci..

[31] Araya-Sibaja A.M., Vega-Baudrit J.R., Guillén-Girón T., Navarro-Hoyos M., Cuffini S.L. (2019). Drug solubility enhancement through the preparation of multicomponent organic materials: Eutectics of lovastatin with carboxylic acids. Pharmaceutics.

[32] Dengale S.J., Grohganz H., Rades T., Löbmann K. (2016). Recent advances in co-amorphous drug formulations. Adv. Drug Deliv. Rev..

[33] Kasten G., Grohganz H., Rades T., Löbmann K. (2016). Development of a screening method for co-amorphous formulations of drugs and amino acids. Eur. J. Pharm. Sci..

[34] Chavan R.B., Thipparaboina R., Kumar D., Shastri N.R. (2016). Co amorphous systems: A product development perspective. Int. J. Pharm..

[35] Laitinen R., Löbmann K., Grohganz H., Priemel P., Strachan C.J., Rades T. (2017). Supersaturating drug delivery systems: The potential of co-amorphous drug formulations. Int. J. Pharm..

[36] Grohganz H., Löbmann K., Priemel P., Tarp Jensen K., Graeser K., Strachan C., Rades T. (2013). Amorphous drugs and dosage forms. J. Drug Deliv. Sci. Technol..

[37] Shi Q., Moinuddin S.M., Cai T. (2019). Advances in coamorphous drug delivery systems. Acta Pharm. Sin. B.

[38] Karagianni A., Kachrimanis K., Nikolakakis I. (2018). Co-Amorphous Solid Dispersions for Solubility and Absorption Improvement of Drugs: Composition, Preparation, Characterization and Formulations for Oral Delivery. Pharmaceutics.

[39] Germán Acacio J.M., Morales-Morales D. (2017). Combination Therapy Including Metformin hydrochloride for the Treatment of Diabetes and Related Comorbidity: A Patent Filed Survey. Org. Med. Chem. Int. J..

[40] Charpentier G. (2002). Oral combination therapy for type 2 diabetes. Diabetes Metab. Res. Rev..

[41] Germán-Acacio J.M., Meza-Sánchez D.E., Morales-Morales D., Atta-ur-Rahman B.T. (2020). Chapter 3—Therapeutically Relevant Natural Products as AMPK Activators in the Treatment of Diabetes. Bioactive Natural Products.

[42] Qaseem A., Humphrey L.L., Sweet D.E., Starkey M., Shekelle P. (2012). Oral pharmacologic treatment of type 2 diabetes mellitus: A clinical practice guideline from the american college of physicians. Ann. Intern. Med..

[43] Sternlicht H., Bakris G.L. (2016). Hydrochlorothiazide as the Diuretic of Choice for Hypertension Time to Kick the Habit. J. Am. Coll. Cardiol..

[44] Engberink R.H.G.O., Frenkel W.J., Van Den Bogaard B., Brewster L.M., Vogt L., Van Den Born B.J.H. (2015). Effects of thiazide-type and thiazide-like diuretics on cardiovascular events and mortality: Systematic review and meta-analysis. Hypertension.

[45] Ong K.L., Barter P.J., Waters D.D. (2014). Cardiovascular drugs that increase the risk of new-onset diabetes. Am. Heart J..

[46] Babu V.R., Hosamani K.M., Aminabhavi T.M. (2008). Preparation and in-vitro release of chlorothiazide novel pH-sensitive chitosan-N,N′-dimethylacrylamide semi-interpenetrating network microspheres. Carbohydr. Polym..

[47] Sanphui P., Rajput L. (2014). Tuning solubility and stability of hydrochloro-thiazide co-crystals. Acta Crystallogr. Sect. B Struct. Sci. Cryst. Eng. Mater..

[48] Wang J.-R., Ye C., Mei X. (2014). Structural and physicochemical aspects of hydrochlorothiazide co-crystals. CrystEngComm.

[49] Sanphui P., Devi V.K., Clara D., Malviya N., Ganguly S., Desiraju G.R. (2015). Cocrystals of hydrochlorothiazide: Solubility and diffusion/permeability enhancements through drug-coformer interactions. Mol. Pharm..

[50] Ghadi R., Dand N. (2017). BCS class IV drugs: Highly notorious candidates for formulation development. J. Control. Release.

[51] Paluch K.J., Tajber L., McCabe T., O’Brien J.E., Corrigan O.I., Healy A.M. (2010). Preparation and solid state characterisation of chlorothiazide sodium intermolecular self-assembly suprastructure. Eur. J. Pharm. Sci..

[52] Paluch K.J., Tajber L., McCabe T., O’Brien J.E., Corrigan O.I., Healy A.M. (2011). Preparation and characterisation of novel chlorothiazide potassium solid-state salt forms: Intermolecular self assembly suprastructures. Eur. J. Pharm. Sci..

[53] Rodrigues M., Lopes J., Sarraguça M. (2018). Vibrational Spectroscopy for Cocrystals Screening. A Comparative Study. Molecules.

[54] Ranjan S., Devarapalli R., Kundu S., Vangala V.R., Ghosh A., Reddy C.M. (2017). Three new hydrochlorothiazide cocrystals: Structural analyses and solubility studies. J. Mol. Struct..

[55] Gopi S.P., Banik M., Desiraju G.R. (2017). New Cocrystals of Hydrochlorothiazide: Optimizing Solubility and Membrane Diffusivity. Cryst. Growth Des..

[56] Aljohani M., Pallipurath A.R., McArdle P., Erxleben A. (2017). A Comprehensive Cocrystal Screening Study of Chlorothiazide. Cryst. Growth Des..

[57] Aljohani M., MacFhionnghaile P., McArdle P., Erxleben A. (2019). Investigation of the formation of drug-drug cocrystals and coamorphous systems of the antidiabetic drug gliclazide. Int. J. Pharm..

[58] Moinuddin S.M., Ruan S., Huang Y., Gao Q., Shi Q., Cai B., Cai T. (2017). Facile formation of co-amorphous atenolol and hydrochlorothiazide mixtures via cryogenic-milling: Enhanced physical stability, dissolution and pharmacokinetic profile. Int. J. Pharm..

[59] Cheng C.L., Yu L.X., Lee H.L., Yang C.Y., Lue C.S., Chou C.H. (2004). Biowaiver extension potential to BCS Class III high solubility-low permeability drugs: Bridging evidence for metformin immediate-release tablet. Eur. J. Pharm. Sci..

[60] Friščič T., Jones W. (2009). Recent advances in understanding the mechanism of cocrystal formation via grinding. Cryst. Growth Des..

[61] James S.L., Adams C.J., Bolm C., Braga D., Collier P., Friščić T., Grepioni F., Harris K.D.M., Hyett G., Jones W. (2012). Mechanochemistry: Opportunities for new and cleaner synthesis. Chem. Soc. Rev..

[62] Braga D., Maini L., Grepioni F. (2013). Mechanochemical preparation of co-crystals. Chem. Soc. Rev..

[63] Tan D., Loots L., Friščić T. (2016). Towards medicinal mechanochemistry: Evolution of milling from pharmaceutical solid form screening to the synthesis of active pharmaceutical ingredients (APIs). Chem. Commun..

[64] Solares-Briones M., Coyote-Dotor G., Páez-Franco J.C., Zermeño-Ortega M.R., de la O Contreras C.M., Canseco-González D., Avila-Sorrosa A., Morales-Morales D., Germán-Acacio J.M. (2021). Mechanochemistry: A Green Approach in the Preparation of Pharmaceutical Cocrystals. Pharmaceutics.

[65] Rycerz L. (2013). Practical remarks concerning phase diagrams determination on the basis of differential scanning calorimetry measurements. J. Therm. Anal. Calorim..

[66] Rodríguez-Carvajal J. (1993). Recent advances in magnetic structure determination by neutron powder diffraction. Phys. B Condens. Matter.

[67] Allen F.H. (2002). The Cambridge Structural Database: A quarter of a million crystal structures and rising. Acta Crystallogr. Sect. B Struct. Sci..

[68] Childs S.L., Chyall L.J., Dunlap J.T., Coates D.A., Stahly B.C., Stahly G.P. (2004). A metastable polymorph of metformin hydrochloride: Isolation and characterization using capillary crystallization and thermal microscopy techniques. Cryst. Growth Des..

[69] Fernandes P., Shankland K., David W.I.F., Markvardsen A.J., Florence A.J., Shankland N., Leech C.K. (2008). A differential thermal expansion approach to crystal structure determination from powder diffraction data. J. Appl. Crystallogr..

[70] Dupont L., Dideberg O. (1972). Structure cristalline de l’hydrochlorothiazide, C7H8ClN3O4S2. Acta Crystallogr. Sect. B.

[71] Bretnall A.E., Clarke G.S., Brittain H.G. (1998). Metformin Hydrochloride. Analytical Profiles of Drug Substances and Excipients.

[72] Clement B., Girreser U. (1999). Characterization of biguanides by 15 N NMR spectroscopy. Magn. Reson. Chem. MRC.

[73] Plata-Vargas E., De la Cruz-Hernández C., Dorazco-González A., Fuentes-Noriega I., Morales-Morales D., Germán-Acacio J.M. (2017). Synthesis of metforminium succinate by melting. Crystal structure, thermal, spectroscopic and dissolution properties. J. Mex. Chem. Soc..

[74] Jakobsen P., Treppendahl S. (1979). The structure of 1,2,4-benzothiadiazine-1,1-dioxides. Tetrahedron.

[75] Brittain H.G. (1990). Chlorothiazide. Analytical Profiles of Drug Substances.

[76] Latosińska J.N. (2003). 13C CP/MAS NMR and DFT studies of thiazides. J. Mol. Struct..

[77] Deppeler H.P. (1981). Hydrochlorothiazide. Anal. Profiles Drug Subst..

[78] Harmon P.A., Yin W., Bowen W.E., Tyrrell R.J., Reed R.A. (2000). Liquid chromatography-mass spectrometry and proton nuclear magnetic resonance characterization of trace level condensation products formed between lactose and the amine-containing diuretic hydrochlorothiazide. J. Pharm. Sci..

[79] Hasa D., Jones W. (2017). Screening for new pharmaceutical solid forms using mechanochemistry: A practical guide. Adv. Drug Deliv. Rev..

[80] Pindelska E., Sokal A., Kolodziejski W. (2017). Pharmaceutical cocrystals, salts and polymorphs: Advanced characterization techniques. Adv. Drug Deliv. Rev..

[81] Latosińska J.N., Latosińska M., Utrecht R., Mielcarek S., Pietrzak J. (2004). Molecular dynamics of solid benzothiadiazine derivatives (Thiazides). A study by NMR, DTA and DFT methods. J. Mol. Struct..

[82] Berry D.J., Steed J.W. (2017). Pharmaceutical cocrystals, salts and multicomponent systems; intermolecular interactions and property based design. Adv. Drug Deliv. Rev..

[83] Figueirêdo C.B.M., Nadvorny D., de Medeiros Vieira A.C.Q., Sobrinho J.L.S., Neto P.J.R., Lee P.I., de La Roca Soares M.F. (2017). Enhancement of dissolution rate through eutectic mixture and solid solution of posaconazole and benznidazole. Int. J. Pharm..

[84] Yadav J.P.A., Bansal A.K., Jain S. (2018). Molecular Understanding and Implication of Structural Integrity in the Deformation Behavior of Binary Drug-Drug Eutectic Systems. Mol. Pharm..

[85] Rajbongshi T., Sarmah K.K., Sarkar A., Ganduri R., Cherukuvada S., Thakur T.S., Thakuria R. (2018). Preparation of Pyrazinamide Eutectics versus Cocrystals Based on Supramolecular Synthon Variations. Cryst. Growth Des..

[86] Patrick Stahly G. (2009). A survey of cocrystals reported prior to 2000. Cryst. Growth Des..

[87] Sarecka-Hujar B., Balwierz R., Ostrozka-Cieslik A., Dyja R., Lukowiec D., Jankowski A. (2017). Scanning electron microscopy and X-ray energy dispersive spectroscopy-useful tools in the analysis of pharmaceutical products. J. Phys. Conf. Ser..

[88] Scoutaris N., Vithani K., Slipper I., Chowdhry B., Douroumis D. (2014). SEM/EDX and confocal Raman microscopy as complementary tools for the characterization of pharmaceutical tablets. Int. J. Pharm..

[89] Newbury D.E. (2009). Mistakes encountered during automatic peak identification of minor and trace constituents in electron-excited energy dispersive X-ray microanalysis. Scanning.

[90] Jain H., Khomane K.S., Bansal A.K. (2014). Implication of microstructure on the mechanical behaviour of an aspirin-paracetamol eutectic mixture. CrystEngComm.

[91] Moore M.D., Wildfong P.L.D. (2009). Aqueous solubility enhancement through engineering of binary solid composites: Pharmaceutical applications. J. Pharm. Innov..

[92] Graeser K.A., Patterson J.E., Zeitler J.A., Rades T. (2010). The role of configurational entropy in amorphous systems. Pharmaceutics.

[93] Meltzer V., Pincu E. (2012). Thermodynamic study of binary mixture of citric acid and tartaric acid. Cent. Eur. J. Chem..

[94] Mahlin D., Bergström C.A.S. (2013). Early drug development predictions of glass-forming ability and physical stability of drugs. Eur. J. Pharm. Sci..

[95] Good D.J., Rodríguez-Hornedo N. (2009). Solubility Advantage of Pharmaceutical Cocrystals. Cryst. Growth Des..

[96] Batisai E., Ayamine A., Kilinkissa O.E.Y., Báthori N.B. (2014). Melting point-solubility-structure correlations in multicomponent crystals containing fumaric or adipic acid. Cryst. Eng. Comm..

[97] Aulton M.E. (2002). Pharmaceutics: The Science of Dosage Form Design.

[98] Baka E., Comer J.E.A., Takács-Novák K. (2008). Study of equilibrium solubility measurement by saturation shake-flask method using hydrochlorothiazide as model compound. J. Pharm. Biomed. Anal..

[99] Goud N.R., Suresh K., Sanphui P., Nangia A. (2012). Fast dissolving eutectic compositions of curcumin. Int. J. Pharm..

[100] Cherukuvada S. (2016). On the issues of resolving a low melting combination as a definite eutectic or an elusive cocrystal: A critical evaluation. J. Chem. Sci..

