# Supplementary material for: Synthesis, Characterization, and Intrinsic Dissolution Studies of Drug–Drug Eutectic Solid Forms of Metformin Hydrochloride and Thiazide Diuretics
Source: Pharmaceutics. 2021 Nov 14;13(11):1926. doi: 10.3390/pharmaceutics13111926 (PMC8620433; doi:10.3390/pharmaceutics13111926)
Supplement: Supplementary file 1 [file pharmaceutics-13-01926-s001.zip › pharmaceutics-1311490-supplementary.pdf]

Supplementary Materials: Synthesis, Characterization, and Intrinsic Dissolution Studies of Drug-Drug Eutectic Solid Forms of Metformin Hydrochloride and Thiazide Diuretics.

Guadalupe Coyote-Dotor, José C. Páez-Franco, Daniel Canseco-González, Alejandra Núñez-Pineda, Alejandro Dorazco-González, Inés Fuentes-Noriega, Alfredo R. Vilchis-Néstor, Joelis Rodríguez Hernández, David Morales-Morales and Juan Manuel Germán-Acacio

Table S1. Rietveld refinements (by NG or LAG solvent-screening) for MET·HCl-CTZ 1:1.

Outcome Solvent-Screening	CTZ	MET·HCl	R_{exp}	R_{wp}	R_B	χ^2
MET·HCl-CTZ 1:1 hexane	68%<br>P1<br>a = 4.8781(6)<br>b = 6.4319(8)<br>c = 8.9939(14)<br>α = 73.901(7)<br>β = 83.704(5)<br>γ = 80.659(5)	32%<br>$P2_1/c$<br>a = 8.0136(9)<br>b = 13.953(1)<br>c = 7.9932(9)<br>β = 114.956(4)	2.86	5.04	3.83	3.10
MET·HCl-CTZ 1:1 acetone	69%<br>P1<br>a = 4.8786(4)<br>b = 6.4251(6)<br>c = 8.9909(9)<br>α = 73.960(6)<br>β = 83.688(5)<br>γ = 80.580(4)	31%<br>$P2_1/c$<br>a = 8.0125(7)<br>b = 13.9509(12)<br>c = 7.9915(6)<br>β = 114.965(5)	3.11	4.83	3.61	2.41
MET·HCl-CTZ 1:1 acetonitrile	70%<br>P1<br>a = 4.8712(4)<br>b = 6.4183(6)<br>c = 8.9857(8)<br>α = 73.944(5)<br>β = 83.637(5)<br>γ = 80.531(4)	30%<br>$P2_1/c$<br>a = 8.0048(6)<br>b = 13.9285(11)<br>c = 7.9798(5)<br>β = 114.998(5)	2.40	3.98	2.98	2.76
MET·HCl-CTZ 1:1 water	67%<br>P1<br>a = 4.8700(4)<br>b = 6.4162(6)<br>c = 8.9838(9)<br>α = 73.942(6)<br>β = 83.681(5)<br>γ = 80.554(5)	33%<br>$P2_1/c$<br>a = 8.0004(6)<br>b = 13.9307(11)<br>c = 7.9780(5)<br>β = 114.957(5)	2.82	4.87	3.55	2.99
MET·HCl-CTZ 1:1 NG	69%<br>P1<br>a = 4.8744(6)<br>b = 6.4269(8)<br>c = 8.9916(13)<br>α = 73.884(6)<br>β = 83.672(6)<br>γ = 80.632(5)	31%<br>$P2_1/c$<br>a = 8.0073(9)<br>b = 13.941(1)<br>c = 7.9865(9)<br>β = 114.956(3)	3.26	4.31	3.20	1.75

Table S2. Rietveld refinements (by NG or LAG solvent-screening) for MET·HCl-HTZ 1:1.

Outcome Solvent-Screening.	HTZ	MET·HCl	R_{exp}	R_{wp}	R_B	χ^2
MET·HCl-HTZ 1:1 hexane	68%<br>$P2_1$<br>a = 7.3984(3)<br>b = 8.5000(3)<br>c = 9.9990(3)<br>β = 111.705(3)	32%<br>$P2_1/c$<br>a = 8.0127(4)<br>b = 13.9483(6)<br>c = 7.9868(3)<br>β = 114.979(3)	3.05	5.31	3.83	3.02
MET·HCl-HTZ 1:1 acetone	67%<br>$P2_1$<br>a = 7.3961(4)<br>b = 8.5004(4)<br>c = 10.0061(6)<br>β = 111.828(8)	33%<br>$P2_1/c$<br>a = 8.0106(5)<br>b = 13.9461(9)<br>c = 7.9858(4)<br>β = 114.970(3)	2.96	5.49	3.96	3.44
MET·HCl-HTZ 1:1 acetonitrile	67%<br>$P2_1$<br>a = 7.3991(3)<br>b = 8.5007(3)<br>c = 10.0087(5)<br>β = 111.839(6)	33%<br>$P2_1/c$<br>a = 8.0147(4)<br>b = 13.9490(7)<br>c = 7.9884(3)<br>β = 114.982(3)	2.40	3.83	2.87	2.54
MET·HCl-HTZ 1:1 water	66%<br>$P2_1$<br>a = 7.3778(6)<br>b = 8.4830(6)<br>c = 9.9885(7)<br>β = 111.866(8)	34%<br>$P2_1/c$<br>a = 8.0007(7)<br>b = 13.928(1)<br>c = 7.9686(5)<br>β = 114.943(4)	2.89	6.60	4.63	5.22
MET·HCl-HTZ 1:1 NG	66%<br>$P2_1$<br>a = 7.4039(5)<br>b = 8.4985(5)<br>c = 10.0092(8)<br>β = 111.82(1)	34%<br>$P2_1/c$<br>a = 8.0168(6)<br>b = 13.947(1)<br>c = 7.9903(4)<br>β = 114.921(4)	2.78	6.22	4.34	5.01

Table S3. Thermodynamic parameters for the construction of the binary phase and Tammann's triangle diagram for MET·HCl-CTZ.

$\chi_{MET·HCl}$	T_{onset} *(1st DSC peak) (°C)* **Solidus**	ΔH_{fus} *(1st DSC peak) (J·g⁻¹)* **Solidus**	T_{onset} *(2nd DSC peak) (°C) Liquidus*	*Heating rate* **(°C/min)**
0.00	360	-	351.49	10
0.17	195.21	36.74	236.12	5
0.20	*195.32*	33.87	231.83	5
0.25	*195.49*	53.99	232.74	5
0.33	*193.25*	55.49	202.81	2
0.50	198.78	163.3	198.78	5
0.66	196.11	194.7	205.80	2
0.75	193.63	78.17	201.20	5
0.80	183.3	53.39	213.19	10
0.83	191.33	44.15	204.14	5
1.00	227.98	-	224.52	10

Table S4. Thermodynamic parameters for the construction of the binary phase and Tammann's triangle diagram for MET·HCl-HTZ.

$\chi_{MET \cdot HCl}$	T_{onset} *(1st DSC peak)* *(°C)* **Solidus**	ΔH_{fus} *(1st DSC peak)* *(J·g^{-1})* **Solidus**	T_{onset} *(2nd DSC peak)* *(°C)* ***Liquidus***	***Heating rate*** **(°C/min)**
0.00	266.2	-	261.05	10
0.17	168.8	30.96	202.9	10
0.20	*166.0*	30.11	200.3	10
0.25	*166.7*	48.73	185.06	10
0.33	*173.4*	69.03	189.67	10
0.50	169.9	122	169.9	10
0.66	174.5	141.4	190.51	10
0.75	167.6	59.59	186.68	10
0.80	170.4	56.42	192.52	10
0.83	167.7	36.99	198.2	10
1.00	227.98	-	224.52	10

Table S5. Rietveld refinements for the different compositions for the solid form MET·HCl-CTZ.

	**METHCl P2$_1$/c**		**CTZ P1**		
METHCl-CTZ<br>0.16: 0.84	a= 8.020(5)Å<br>b=13.964(4)Å<br>c= 7.993(5)Å<br>β= 115.08(2)º	7%<br>V= 810.7(2) Å^3	a= 4.887(2)Å<br>b= 6.428(3)Å<br>c= 9.009(2)Å<br>α= 73.92(2)º<br>β= 83.54(2)º<br>γ= 80.56(2)º	93%<br>V= 267.64(7) Å^3	R_{exp}= 6.40<br>R_B= 8.13<br>R_{wp}= 11.0<br>χ^2= 2.95
METHCl-CTZ<br>0.20:0.80	a= 8.026(5) Å<br>b=13.965(9) Å<br>c= 7.994(4) Å<br>β= 115.09(5) º	8%<br>V= 811.4(9)Å^3	a= 4.895(1) Å<br>b= 6.431(1) Å<br>c= 9.016(2) Å<br>α= 73.96(1) º<br>β= 83.55(1) º<br>γ= 80.56(1) º	92%<br>V= 268.45(8)Å^3	R_{exp}= 8.17<br>R_B= 8.48<br>R_{wp}= 11.6<br>χ^2= 2.02
METHCl-CTZ<br>0.25:0.75	a= 8.019(4) Å<br>b=13.963(7) Å<br>c= 7.990(3) Å<br>β= 115.07(4) º	12%<br>V= 810.3(6)Å^3	a= 4.889(1) Å<br>b= 6.427(1) Å<br>c= 9.013(2) Å<br>α= 73.93(1) º<br>β= 83.54(1) º<br>γ= 80.57(1) º	88%<br>V= 267.76(9)Å^3	R_{exp}= 7.77<br>R_B= 8.67<br>R_{wp}= 11.7<br>χ^2= 2.28
METHCl-CTZ<br>0.33:0.66	a= 8.028(4)Å<br>b=13.967(7)Å<br>c= 7.998(3)Å<br>β= 115.04(3)º	16%<br>V= 812.4(6)Å^3	a= 4.890(1) Å<br>b= 6.429(1) Å<br>c= 9.015(2) Å<br>α= 73.95(1) º<br>β= 83.56(1) º<br>γ= 80.59(1) º	84%<br>V= 268.1(1)Å^3	R_{exp}= 7.27<br>R_B= 8.37<br>R_{wp}= 10.9<br>χ^2= 2.26
METHCl-CTZ<br>0.5:0.5	a= 8.032(4)Å<br>b=13.968(7)Å<br>c= 7.990(7)Å<br>β= 115.09(3)º	27%<br>V= 811.8(7) Å^3	a= 4.891(2)Å<br>b= 6.428(2)Å<br>c= 9.012(3)Å<br>α= 73.96(2)º<br>β= 83.54(2)º<br>γ= 80.57(2)º	73%<br>V= 268.0(2) Å^3	R_{exp}= 9.58<br>R_B= 11.6<br>R_{wp}= 15.4<br>χ^2= 2.60
METHCl-CTZ<br>0.66:0.33	a= 8.029(2) Å<br>b=13.975(4) Å<br>c= 7.998(2) Å<br>β= 115.00(2) º	45%<br>V= 813.4(4) Å^3	a= 4.888(2) Å<br>b= 6.430(2) Å<br>c= 9.020(3) Å<br>α= 73.97(2) º<br>β= 83.57(2) º<br>γ= 80.59(2) º	55%<br>V= 268.2(1) Å^3	R_{exp}= 8.40<br>R_B= 8.83<br>R_{wp}= 11.9<br>χ^2= 2.00
METHCl-CTZ	a= 8.026(1) Å	57%	a= 4.890(2) Å	43%	R_{exp}= 6.31

0.75:0.25	b=13.968(3) Å<br>c= 8.000(1) Å<br>β= 115.01(1) º	V= 812.8(3) Å^3	b= 6.429(2) Å<br>c= 9.018(3) Å<br>α= 73.93(2) º<br>β= 83.55(2) º<br>γ= 80.57(2) º	V= 268.1(1) Å^3	R_B= 7.74<br>R_{wp}= 10.8<br>χ^2= 2.92
METHCl-CTZ<br>0.80:0.20	a= 8.020(2) Å<br>b=13.963(3) Å<br>c= 7.999(2) Å<br>β= 115.03(2)º	66%<br>V= 811.6(3) Å^3	a= 4.882(3) Å<br>b= 6.421(2) Å<br>c= 9.015(4) Å<br>α= 73.96(3) º<br>β= 83.49(3) º<br>γ= 80.47(3) º	34%<br>V= 267.2(2) Å^3	R_{exp}= 6.71<br>R_B= 7.47<br>R_{wp}= 10.4<br>χ^2= 2.40
METHCl-CTZ<br>0.84:0.16	a= 8.031(2) Å<br>b=13.978(3) Å<br>c= 8.007(2) Å<br>β= 115.02(1) º	68%<br>V= 814.6(3) Å^3	a= 4.892(3) Å<br>b= 6.429(3) Å<br>c= 9.025(4) Å<br>α= 73.95(3) º<br>β= 83.58(3) º<br>γ= 80.53(3) º	32%<br>V= 268.4(2) Å^3	R_{exp}= 6.75<br>R_B= 9.69<br>R_{wp}= 12.8<br>χ^2= 3.61

Table S6. Rietveld refinements for the different compositions for the solid form MET·HCl-HTZ.

	**METHCl $P2_1/c$**		**HTZ $P2_1$**		
METHCl-HTZ<br>0.16:0.84	a= 8.023(8) Å<br>b=13.955(14) Å<br>c= 7.992(5) Å<br>β= 115.02(7) º	5%<br>V= 811(1) Å^3	a= 7.408(1) Å<br>b= 8.513(1) Å<br>c= 10.014(2) Å<br>β= 111.71(2) º	95%<br>V= 586.7(2) Å^3	R_{exp}= 7.62<br>R_B= 9.32<br>R_{wp}= 12.7<br>χ^2= 2.77
METHCl-HTZ<br>0.20:0.80	a= 8.019(5) Å<br>b=13.969(9) Å<br>c= 7.992(3) Å<br>β= 115.07(5) º	8%<br>V= 810.8(8)Å^3	a= 7.406(1) Å<br>b= 8.505(1) Å<br>c= 10.008(1) Å<br>β= 111.75(1) º	92%<br>V= 585.5(1)Å^3	R_{exp}= 6.60<br>R_B= 7.58<br>R_{wp}= 9.38<br>χ^2= 2.02
METHCl-HTZ<br>0.25:0.75	a= 8.026(5) Å<br>b=13.985(9) Å<br>c= 7.997(4) Å<br>β= 115.06(5) º	10%<br>V= 813.2(9)Å^3	a= 7.410(1) Å<br>b= 8.510(1) Å<br>c= 10.016(2) Å<br>β= 111.72(2) º	90%<br>V= 586.7(2)Å^3	R_{exp}= 7.19<br>R_B= 8.09<br>R_{wp}= 10.7<br>χ^2= 2.22
METHCl-HTZ<br>0.33:0.66	a= 8.015(3) Å<br>b=13.962(6) Å<br>c= 7.992(3) Å<br>β= 115.05(3)º	15%<br>V= 810.2(6)Å^3	a= 7.407(1) Å<br>b= 8.507(1) Å<br>c= 10.009(2) Å<br>β= 111.75(2) º	85%<br>V= 585.7(2)Å^3	R_{exp}= 7.06<br>R_B= 8.00<br>R_{wp}= 11.0<br>χ^2= 2.45
METHCl-HTZ<br>0.50:0.50	a= 8.032(2) Å<br>b=13.975(4) Å<br>c= 8.005(2) Å<br>β= 115.04(2) º	31%<br>V= 814.1(7) Å^3	a= 7.419(2) Å<br>b= 8.518(1) Å<br>c= 10.023(2) Å<br>β= 111.73(2) º	69%<br>V= 588.3(2) Å^3	R_{exp}= 6.68<br>R_B= 7.18<br>R_{wp}= 9.51<br>χ^2= 2.03
METHCl-HTZ<br>0.66:0.33	a= 8.026(2) Å<br>b=13.972(4) Å<br>c= 8.002(2) Å<br>β= 114.99(2) º	47%<br>V= 813.2(4) Å^3	a= 7.410(2) Å<br>b= 8.517(2) Å<br>c= 10.011(3) Å<br>β= 111.72(3) º	53%<br>V= 586.9(3) Å^3	R_{exp}= 8.96<br>R_B= 9.90<br>R_{wp}= 13.3<br>χ^2= 2.21
METHCl-HTZ<br>0.75:0.25	a= 8.022(1) Å<br>b=13.947(2) Å<br>c= 7.990(1) Å<br>β= 115.02(1) º	57%<br>V= 810.1(2) Å^3	a= 7.406(2) Å<br>b= 8.506(1) Å<br>c= 9.990(2) Å<br>β= 111.92(2) º	43%<br>V= 583.8(2) Å^3	R_{exp}= 6.83<br>R_B= 8.66<br>R_{wp}= 11.7<br>χ^2= 2.92
METHCl-HTZ<br>0.80:0.20	a= 8.030(1) Å<br>b=13.968(3) Å<br>c= 8.002(1) Å<br>β= 115.03(1) º	68%<br>V= 813.3(2) Å^3	a= 7.416(3) Å<br>b= 8.511(2) Å<br>c= 10.018(3) Å<br>β= 111.76(4) º	32%<br>V= 587.2(3) Å^3	R_{exp}= 6.41<br>R_B= 9.33<br>R_{wp}= 10.4<br>χ^2= 2.65
METHCl-HTZ<br>0.84:0.16	a= 8.032(1) Å<br>b=13.969(2) Å<br>c= 8.002(1) Å<br>β= 115.02(1) º	68%<br>V= 813.6(2) Å^3	a= 7.415(2) Å<br>b= 8.516(2) Å<br>c= 10.012(3) Å<br>β= 111.72(3) º	32%<br>V= 587.4(2) Å^3	R_{exp}= 6.69<br>R_B= 7.73<br>R_{wp}= 11.0<br>χ^2= 2.71

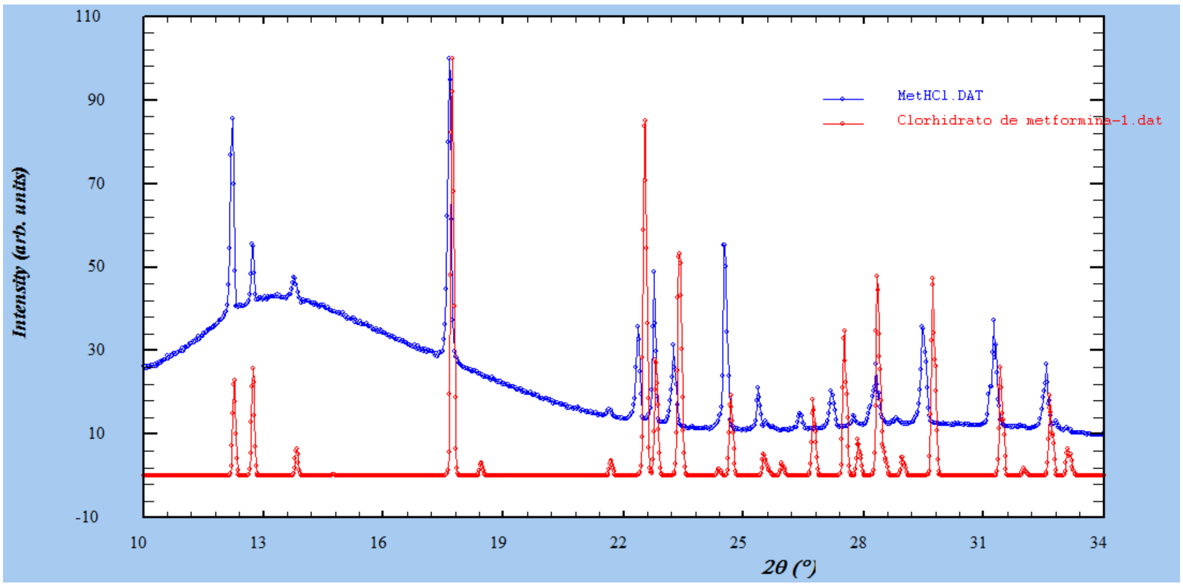

Figure S1. Rietveld refinement JAMRIY01 [68] (MET·HCl polymorph A, red) *vs* pure MET·HCl (in blue) purchased from Tokyo Chemical Industry™.

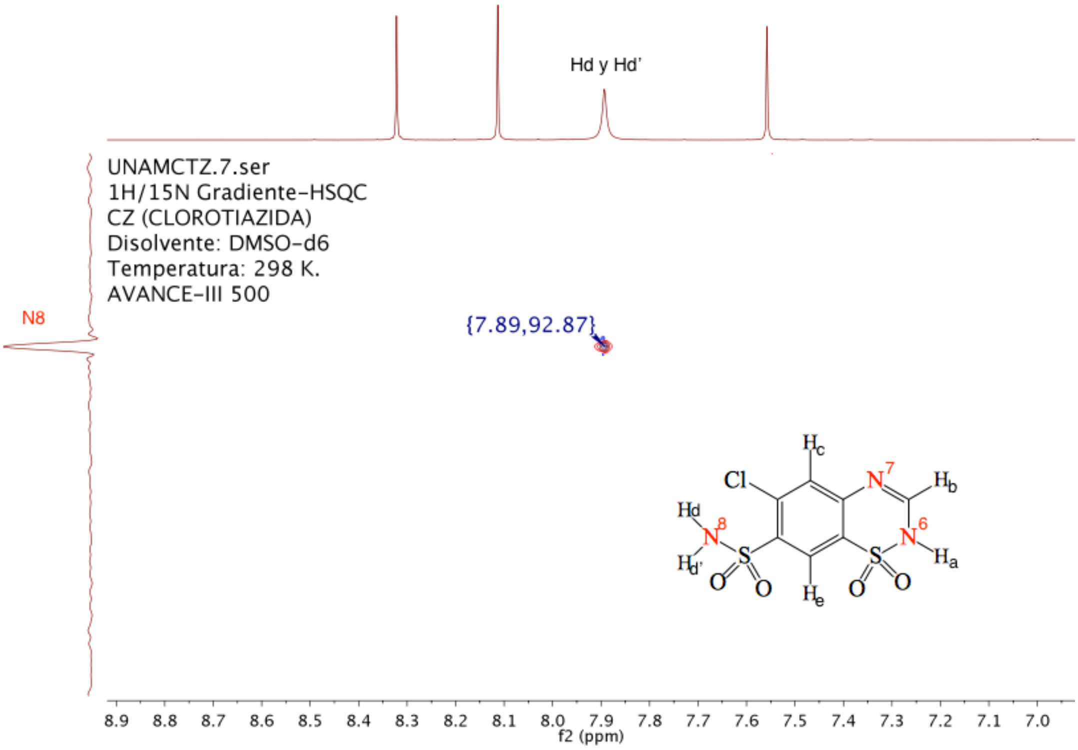

Figure S2. HSQC ^{1}H–^{15}N CTZ in d$_6$-DMSO.

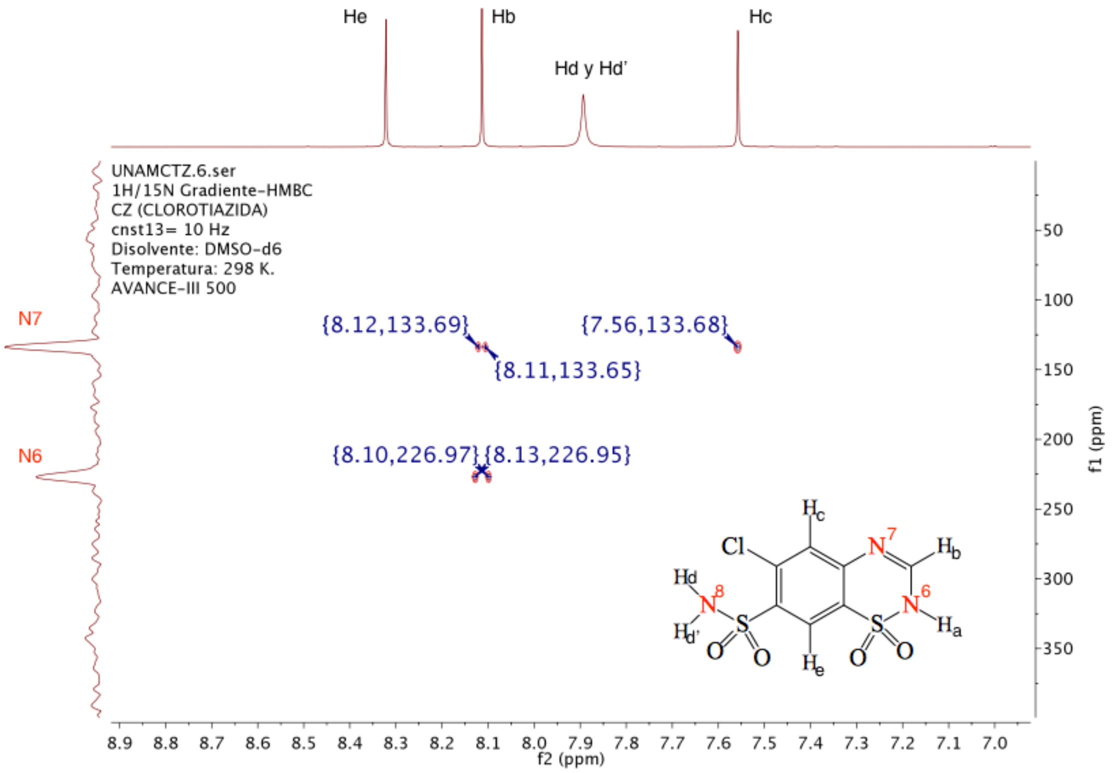

Figure S3. HMBC ^{1}H–^{15}N CTZ in d_6-DMSO.

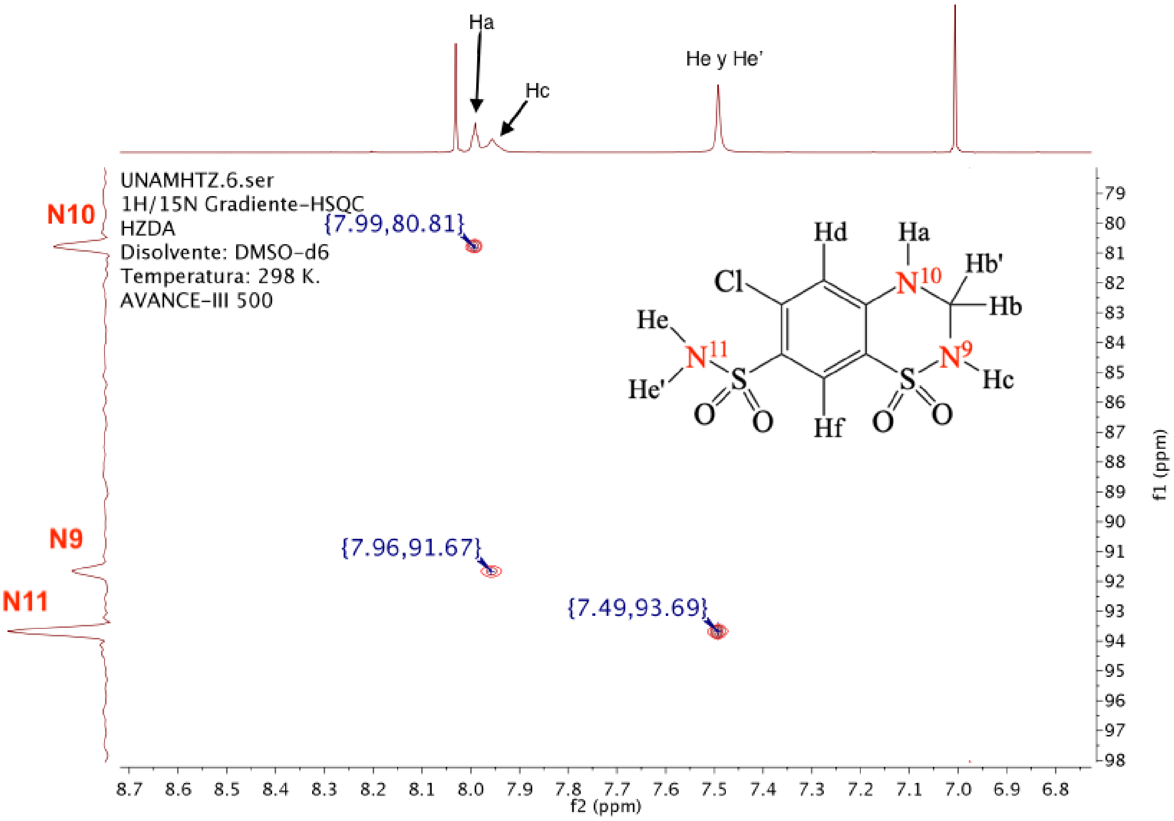

Figure S4. HSQC ^{1}H–^{15}N HTZ in d_6-DMSO.

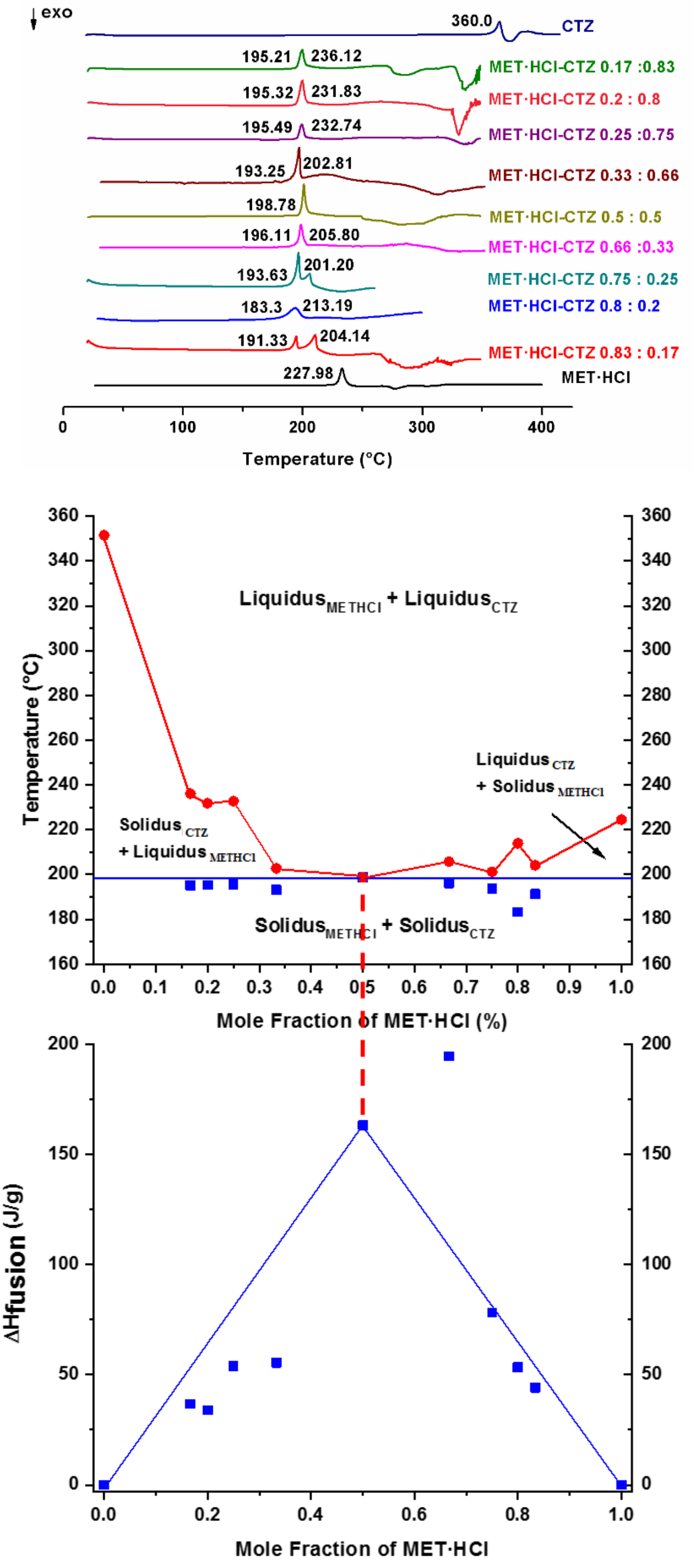

Figure S5. Enlarged images of Figures 9a and 9b.

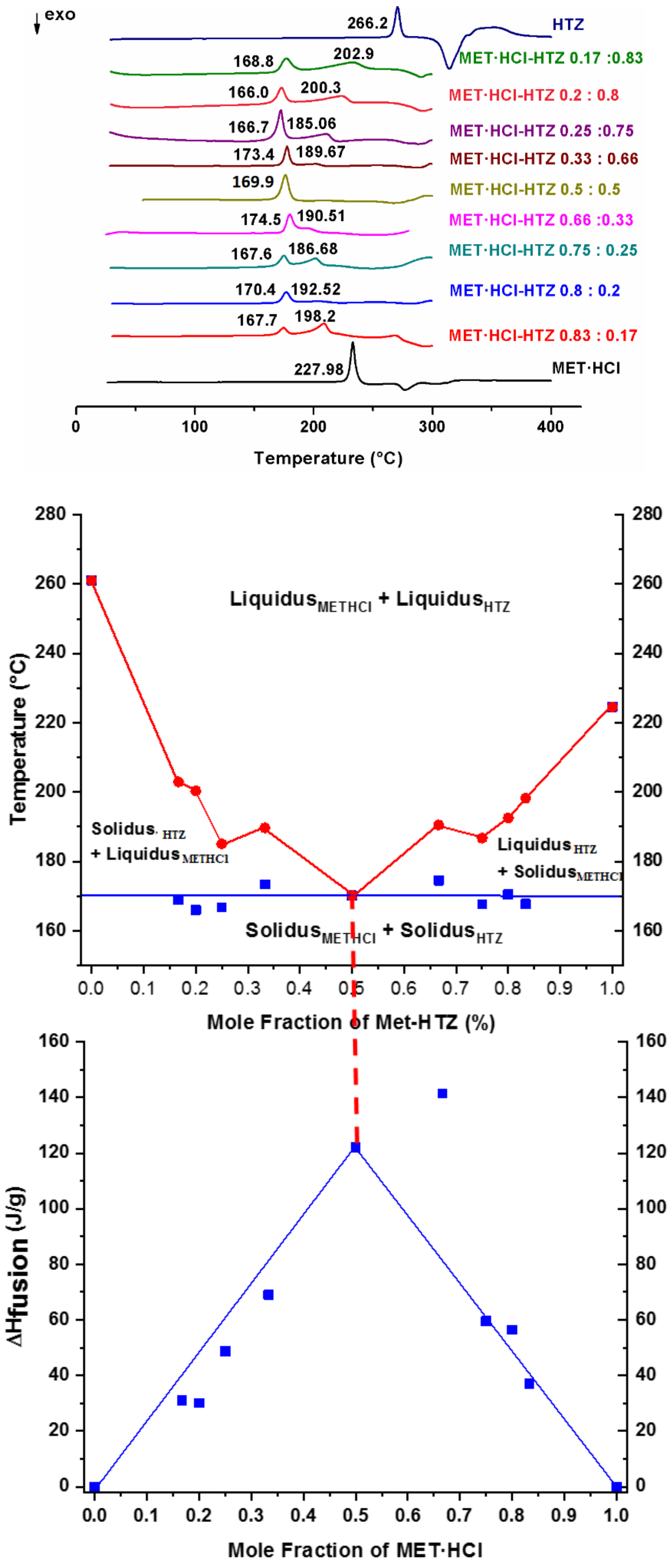

Figure S6. Enlarged images of Figures 10a and 10b.

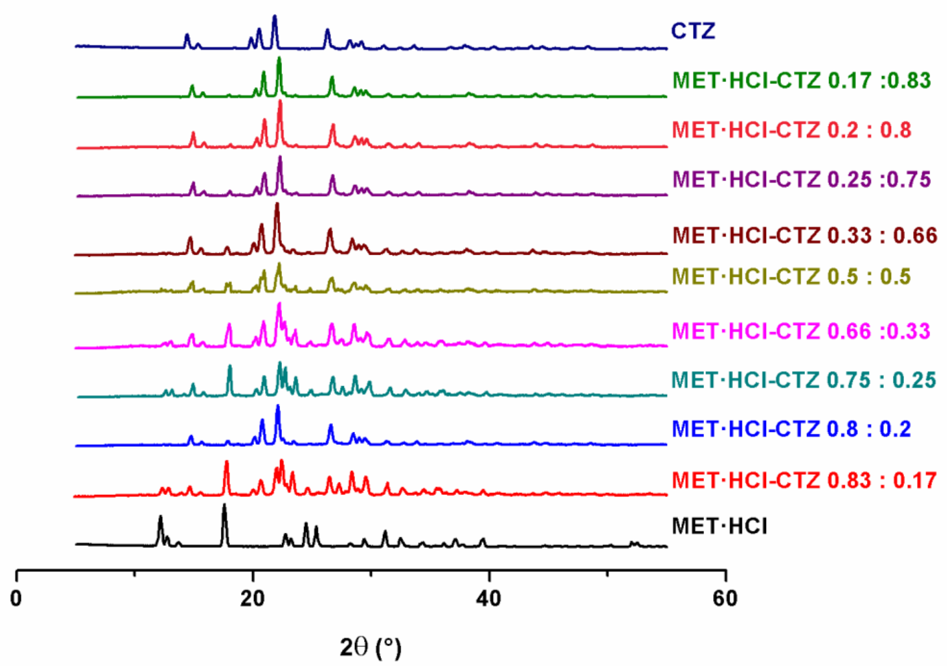

Figure S7. PXRD experiments for the different compositions for the solid form MET·HCl-CTZ.

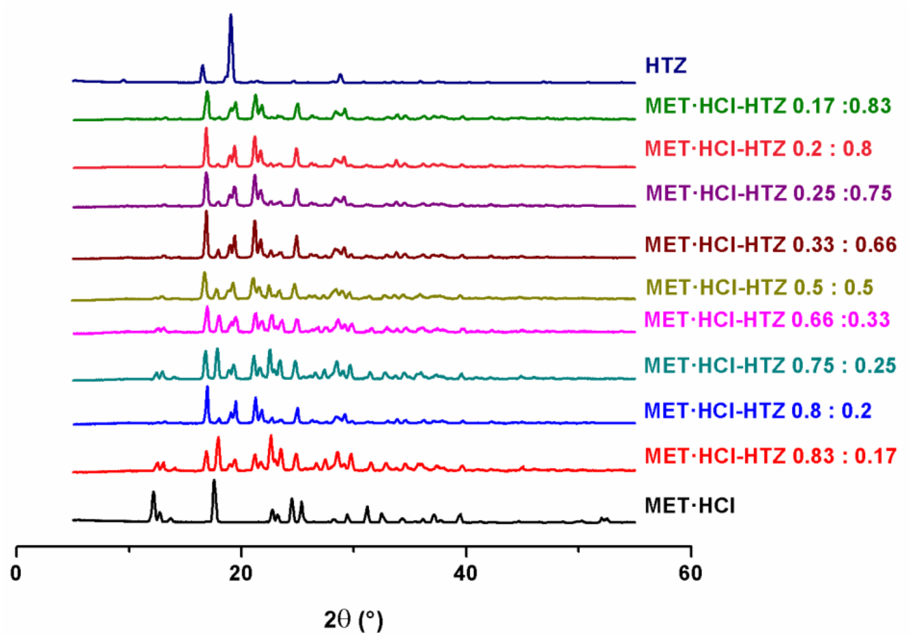

Figure S8. PXRD experiments for the compositions for the solid form MET·HCl-HTZ.

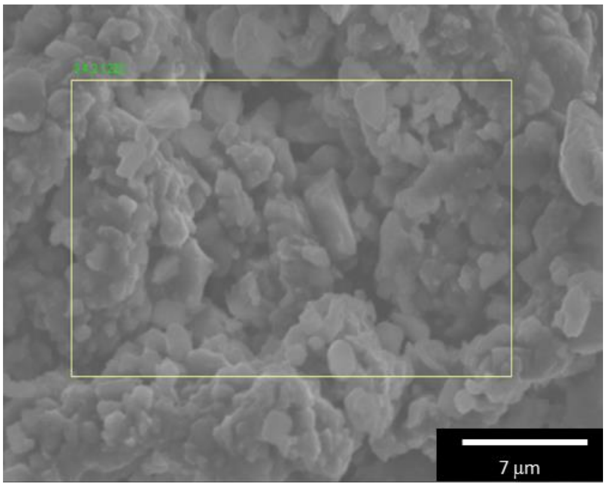

Element	Carbon	Nitrogen	Oxygen	Sulfur	Chlorine
**% Weight**	38.53	25.17	15.81	9.00	11.49

Figure S9. SEM-EDS elemental composition analysis for the solid form MET·HCl-CTZ 1:1 ($\chi_{MET \cdot HCl} = 0.5$).

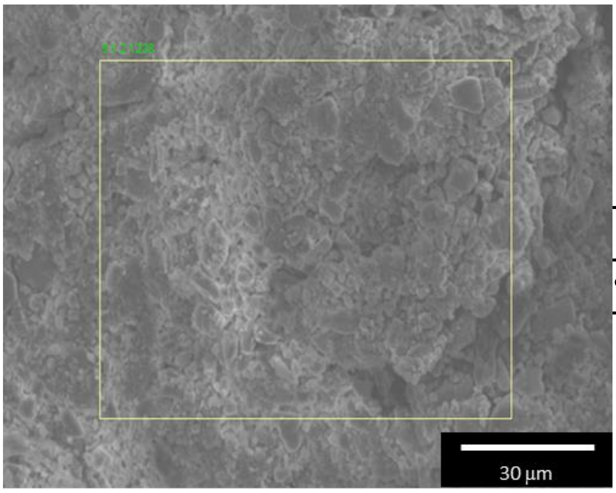

Element	Carbon	Nitrogen	Oxygen	Sulfur	Chlorine
**% Weight**	35.55	19.91	15.09	14.97	14.49

Figure S10. SEM-EDS elemental composition analysis for the solid form MET·HCl-HTZ 1:1 ($\chi_{MET \cdot HCl} = 0.5$).

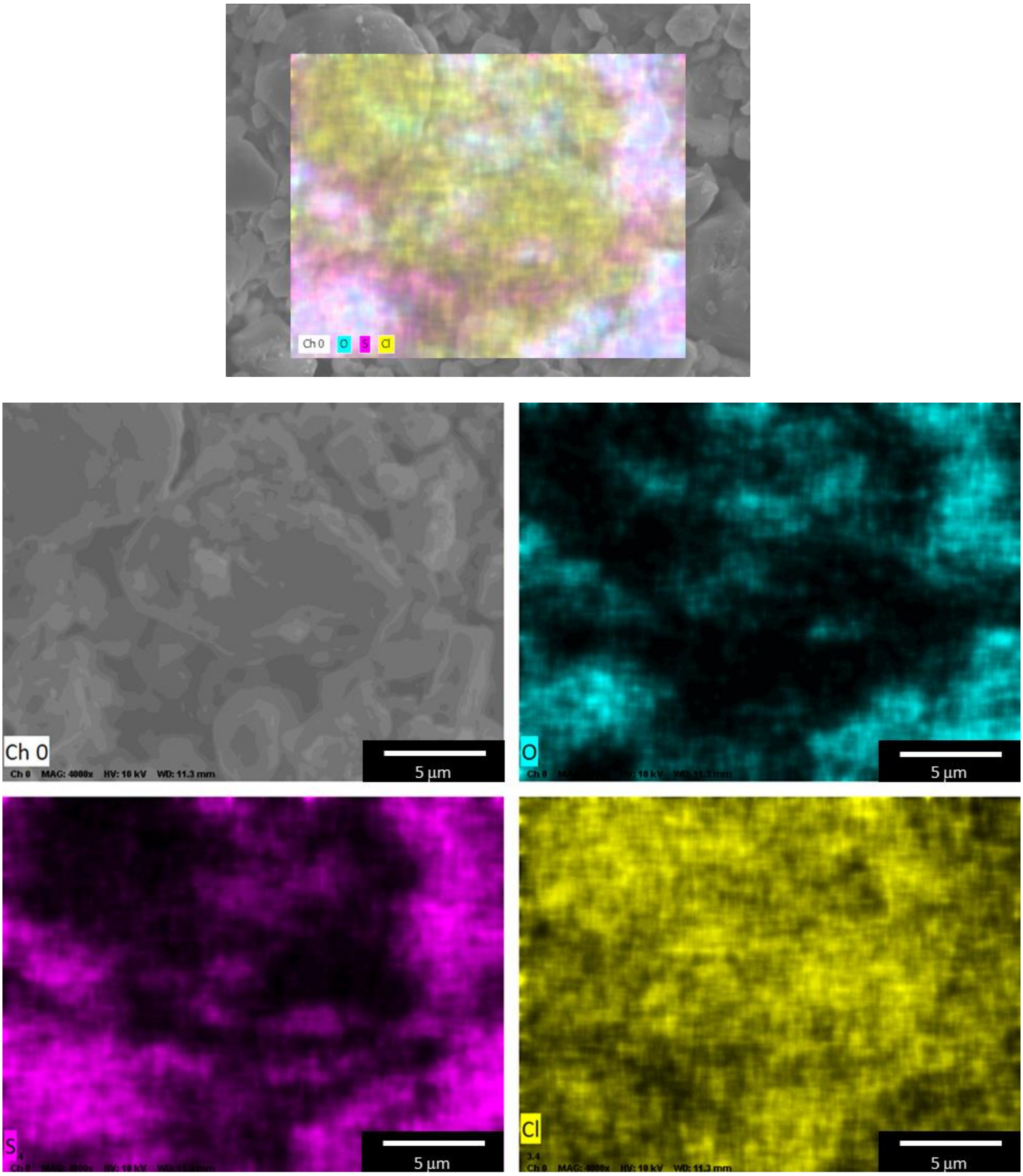

Figure S11. EDS mapping via SEM imagen for MET·HCl-CTZ 1:1 ($\chi_{MET \cdot HCl}$ = 0.5).

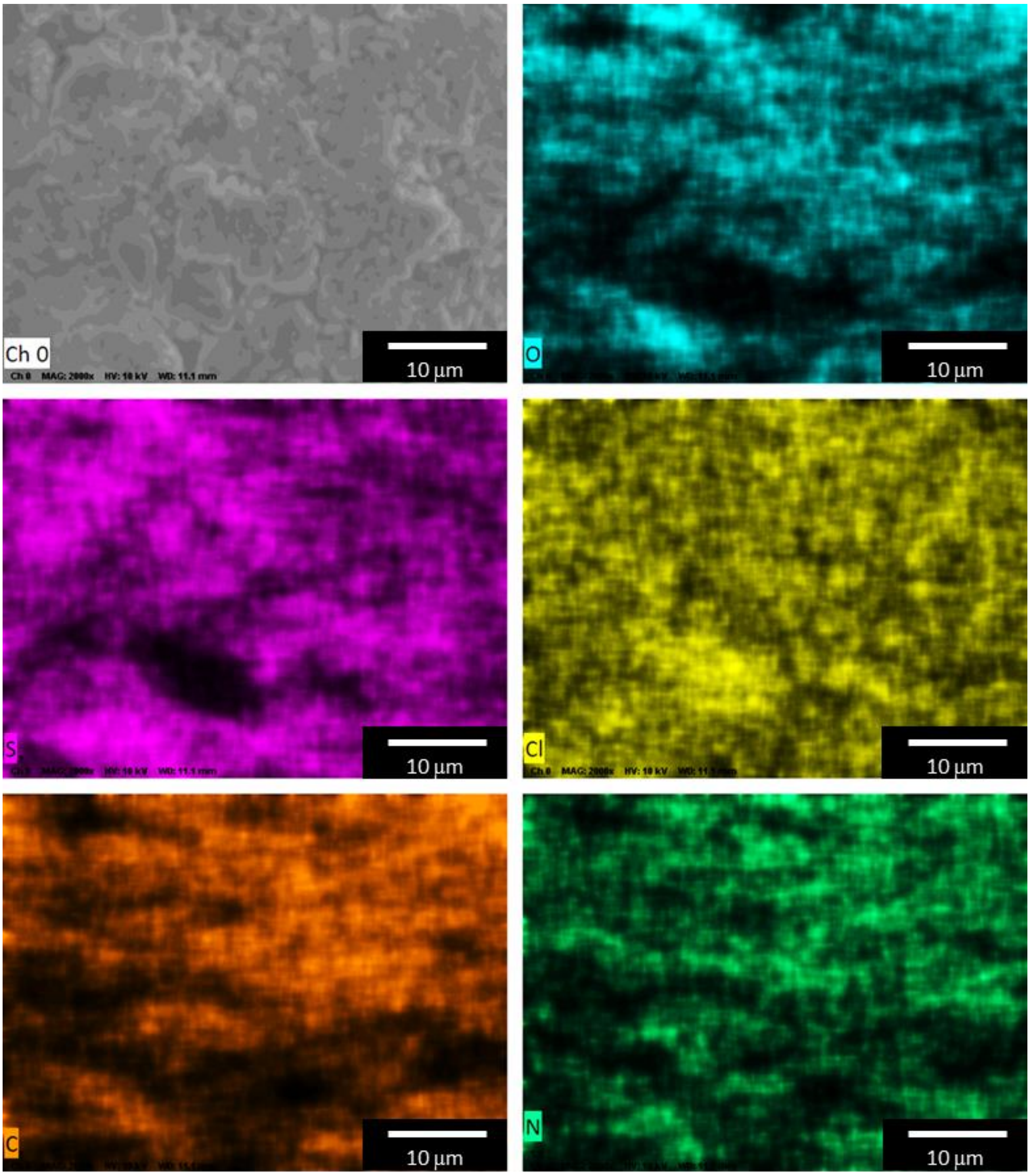

Figure S12. EDS mapping via SEM imagen for MET·HCl-HTZ 1:1 ($\chi_{MET\cdot HCl} = 0.5$).
